# Early Long-Term Memory Impairment and Changes in the Expression of Synaptic Plasticity-Associated Genes, in the McGill-R-Thy1-APP Rat Model of Alzheimer's-Like Brain Amyloidosis

**DOI:** 10.3389/fnagi.2020.585873

**Published:** 2021-01-22

**Authors:** Martín Habif, Sonia Do Carmo, María Verónica Báez, Natalia Claudia Colettis, Magalí Cecilia Cercato, Daniela Alejandra Salas, María Florencia Acutain, Caterina Laura Sister, Valeria Laura Berkowicz, María Pilar Canal, Tomás González Garello, A. Claudio Cuello, Diana Alicia Jerusalinsky

**Affiliations:** ^1^Laboratory of Neuroplasticity and Neurotoxins (LaN&N), Facultad de Medicina, Instituto de Biología Celular y Neurociencia (IBCN) “Prof. Eduardo De Robertis” (Universidad de Buenos Aires – CONICET), Buenos Aires, Argentina; ^2^Department of Pharmacology and Therapeutics, McGill University, Montreal, QC, Canada; ^3^Department of Anatomy and Cell Biology, McGill University, Montreal, QC, Canada; ^4^Department of Neurology and Neurosurgery, McGill University, Montreal, QC, Canada

**Keywords:** amyloid beta-precursor protein, long-term memory, cognitive dysfunction, social behavior alterations, neuronal plasticity, *Camk2b*, *Grin2b*, Alzheimer disease

## Abstract

Accruing evidence supports the hypothesis that memory deficits in early Alzheimer Disease (AD) might be due to synaptic failure caused by accumulation of intracellular amyloid beta (Aβ) oligomers, then secreted to the extracellular media. Transgenic mouse AD models provide valuable information on AD pathology. However, the failure to translate these findings to humans calls for models that better recapitulate the human pathology. McGill-R-Thy1-APP transgenic (Tg) rat expresses the human amyloid precursor protein (APP751) with the Swedish and Indiana mutations (of familial AD), leading to an AD-like slow-progressing brain amyloid pathology. Therefore, it offers a unique opportunity to investigate learning and memory abilities at early stages of AD, when Aβ accumulation is restricted to the intracellular compartment, prior to plaque deposition. Our goal was to further investigate early deficits in memory, particularly long-term memory in McGill-R-Thy1-APP heterozygous (Tg+/–) rats. Short-term- and long-term habituation to an open field were preserved in 3-, 4-, and 6-month-old (Tg+/–). However, long-term memory of inhibitory avoidance to a foot-shock, novel object-recognition and social approaching behavior were seriously impaired in 4-month-old (Tg+/–) male rats, suggesting that they are unable to either consolidate and/or evoke such associative and discriminative memories with aversive, emotional and spatial components. The long-term memory deficits were accompanied by increased transcript levels of genes relevant to synaptic plasticity, learning and memory processing in the hippocampus, such as *Grin2b, Dlg4, Camk2b*, and *Syn1*. Our findings indicate that in addition to the previously well-documented deficits in learning and memory, McGill-R-Thy1-APP rats display particular long-term-memory deficits and deep social behavior alterations at pre-plaque early stages of the pathology. This highlights the importance of Aβ oligomers and emphasizes the validity of the model to study AD-like early processes, with potentially predictive value.

## Introduction

For many years, memory failure in Alzheimer disease (AD) was attributed to neuronal death induced by deposition of insoluble amyloid fibrils. However, accruing evidence supports the hypothesis that memory impairment in early AD might be due to synaptic failure caused by the accumulation of intracellular small soluble amyloid beta (Aβ) oligomers (ADDLs or AβOs)—see (Klein, 2006), then secreted to the extracellular media.

Transgenic (Tg) mouse models of AD have provided valuable information on processes involved in AD pathology. However, the failure to translate these findings to humans calls for the use of models that better recapitulate the human pathology. Rats are genetically and physiologically closer to humans than mice (Do Carmo and Cuello, 2013), namely regarding tau and apoE (Tran et al., 2013). For example, similar to humans, the adult rat brain contains six tau isoforms while the mouse brain only contains three tau isoforms (Hanes et al., 2009; Liu and Götz, 2013). In addition, rats display a larger behavioral repertoire compared to mice, which facilitates more refined cognitive assessments.

The McGill-R-Thy1-APP rat model of AD-like amyloid pathology offers a unique opportunity for testing learning and memory abilities at Aβ intracellular -or earliest extracellular- stages, prior to Aβ deposition as plaques (Leon et al., 2010; Iulita et al., 2014a). McGill-R-Thy1-APP Tg rats (Leon et al., 2010) express the human amyloid precursor protein 751 (APP751) with the Swedish and Indiana mutations, and display a slow-progressing amyloid pathology with intraneuronal human Aβ accumulation (Cuello et al., 2012) starting 1 week after birth and leading to an extracellular amyloid pathology by 6–9-months of age, in homozygous Tg+/+ rats (Leon et al., 2010; Iulita et al., 2014a). The earliest extracellular amyloid plaques are detected sequentially in the subiculum, hippocampus, cerebral cortex and some sub-cortical regions. In contrast, in heterozygous Tg+/– rats, the Aβ pathology is mostly restricted to the intracellular compartment with scarce amyloid plaques appearing at advanced ages.

The progressive amyloid pathology is accompanied by cognitive impairments, which are detectable as early as 3-months of age, prior to the deposition of amyloid plaques. Tg+/+ rats showed significant impairments in learning and memory in several tasks, including long term memory (LTM) in a cued fear conditioning paradigm (Iulita et al., 2014a), as well as short term memory (STM) in new object recognition (NOR) and location (NOL) tasks (Cuello et al., 2012; Galeano et al., 2014; Iulita et al., 2014a) with Tg+/– rats displaying an intermediate performance between Tg+/+ and wt populations (Leon et al., 2010; Iulita et al., 2014a). Leon et al. (2010) also reported that 3-month-old Tg+/+ rats display cognitive impairments in the Morris water Maze (MWM), which measures various aspects of STM and LTM, while Tg+/– were able to learn the task. Accordingly, long-term potentiation (LTP) was strongly impaired in 4-month-old Tg rats, where a 200 Hz (HFS) conditioning protocol failed to induce LTP. A similar significant reduction in the initial magnitude of potentiation (short-term potentiation, STP), as measured in the first 10 min after HFS, was present in 4-month-old Tg rats (Qi et al., 2014).

In an attempt to further elucidate the earliest deficits in memory acquisition and consolidation, particularly in LTM in this rat model of AD-like brain amyloidosis, we evaluated Tg+/– rats performance at different ages in various tasks in comparison to their wt littermates. We assessed short-term—intra-session exploratory behavior—and long-term habituation to an open field (OF), as well as LTM of inhibitory avoidance (IA) to a mild foot-shock. Thereafter, we also assessed object discrimination and social behavior involving novel experience, memory and congeners interaction. Finally, we examined the expression of genes related to synaptic plasticity, learning and memory processing. Our assays were carried out with male rats only, while in most previous reports both sexes were used indiscriminately.

## Materials and Methods

### Animals

McGill-R-Thy1-APP Tg rats (Leon et al., 2010) were obtained originally from Dr. A.C. Cuello's Laboratory (Department of Pharmacology and Therapeutics, McGill University, Montreal, Canada). Then, a colony was established in our animal facility. Tg+/+ and Tg+/– rats, as well as their wild type littermates were obtained by crossbreeding Tg+/+ with Tg+/–, and also by crossbreeding them with wild-type Wistar rats. Adult male rats (200–250 g) were maintained in groups of four to five animals per cage, under a 12 h light-12 h dark inverted cycle at 25°C room temperature and *ad libitum* access to food and water.

Heterozygous Tg+/– rats were identified by PCR amplification of DNA extracted from ear biopsy tissue to assess the presence of the hAβPP transgene (Gene ID: NM_000484.4) using the following primers: *hAPP-Fw:5*′*-AGGACTGACCACTCGACCAG-3*′ and *hAPP-Rv:5*′*-CGGGGGTCTAGTTCTGGAT-3*′*. Rattus norvegicus* peptidylprolyl isomerase G – (Ppig) (Gene ID: XM_006234324.3) was used as a housekeeping control gene, employing the following primers: *CycloFw:5*′*-TACAACAGTAGAACAAGGGAGCGAAG-3*′ and *CycloRv:5*′*-ATCCCTCCTTCTTCTCCTCCTATCTTT-3*′ (Supplementary Figure 1). The same set of primers were used for genotyping homozygous Tg+/+. In this case RNA was extracted from ear biopsy tissue using Trizol (Invitrogen) and used as template for cDNA synthesis, which was then subjected to real-time PCR amplification.

All the experiments were performed in accordance with the School of Medicine Institutional Committee for Care and Use of Laboratory Animals (CICUAL) of the University of Buenos Aires (Argentina), and in compliance with the Guide to the Care and Use of Experimental Animals of the Canadian Council on Animal Care (Canada) and the National Institutes of Health (NIH) (USA).

### Behavioral Tasks

Two sets of behavioral experiments were performed. In the first set 3-, 4-, and 6-month-old male Tg +/– rats and their wt male littermates were trained in several behavioral tasks along 3 weeks. Training and tests protocols started 1 week before the rats were 3-, 4- or 6-months-old and finished when they were 3-, 4-, or 6-months plus 2 weeks. Animals were first left to explore an open field (OF) and then, tested in the same arena. On the following week, rats were trained in a step through-inhibitory avoidance (IA) to a mild foot-shock as aversive cue. Finally, a tail-flick test was carried out to corroborate pain sensitivity in these rats.

In another set of experiments, 4-month-old male Tg+/– rats, as well as their wt littermates were trained and assessed in a novel object recognition (NOR) task adapted to assess long-term memory (LTM). Other groups of 4-month-old male Tg+/+, Tg+/– and their wt littermates were trained and tested for social approaching behavior (SA), in a slightly modified three-chambered task (Yang et al., 2011).

### Open Field Task (OF)

Exploratory activity and habituation to the OF of 3-, 4-, and 6-month-old wt and heterozygous Tg+/– rats were assessed (3 mo: N_wt_ = 8, N_Tg+/−_ = 11; 4 mo: N_wt_ = 8, N_Tg+/−_ = 11; 6 mo: N_wt_ = 11, N_Tg+/−_ = 10). In the first day, each rat was individually exposed to the OF (75.0 cm long × 75.0 cm wide × 50.0 cm high) for 5 min (training session). Immediately after, the animal was returned to its home cage with food and water *ad libitum*. The OF has visual cues on its walls: two opposite walls are plain, and the other two are striped, one with vertical and the other with horizontal stripes. Twenty-five squares (15.0 cm × 15.0 cm) are drawn on the floor. The overall trajectory in the experimental arena was tracked in real-time with *Tracker*© video analysis and modeling tool, from the Open-Source Physics (OSP) Java framework. The resulting .csv files were analyzed with Python 3.6.4 custom made scripts, and data visualized with matplotlib 2D plotting library. Traveled distance—to assess locomotor activity -, resting time and time spent in central squares vs. in corners and peripheral squares close to the walls—to assess fear and anxiety-like behavior -, as well as grooming events—as stereotyped behavior- were quantified along the 5 min first exposure to the OF. The number of crossings across the lines in the floor was recorded as an indication of “horizontal” exploratory activity and was compared within the training session (minute by minute) to evaluate habituation to the environment. The number of *rearings* was taken into account as an indicator of “vertical” exploratory activity. Some rats (3 mo: N_wt_ = 8, N_Tg+/−_ = 8; 4 mo: N_wt_ = 8, N_Tg+/−_ = 9; 6 mo: N_wt_ = 11, N_Tg+/−_ = 8) were tested 24 h later in the same OF to assess LTM and long-term (LT) habituation. Animals were considered habituated to the arena when the total number of crossings (as well as rearings) recorded over 5 min, was significantly lower in the test than in the training session.

### Step Through Inhibitory Avoidance (IA)

For the IA task, an acrylic box (50 cm long × 25 cm wide × 30 cm high) with a grid floor made of parallel bronze bars (0.3 cm in diameter, set 0.5 cm apart) was used; this box was divided into two compartments: a dark one and an enlighten one, separated by a “guillotine door.” During the training session, rats (3 mo: N_wt_ = 8, N_Tg+/−_ = 7; 4 mo: N_wt_ = 7, N_Tg+/−_=11; 6 mo: N_wt_ = 11, N_Tg+/−_ = 13) were left to freely explore the enlighten compartment for 15 s, while the guillotine door remained closed. Then, the door was opened by sliding it up, and the latency to escape from the light to the dark –preferred- side was recorded. The door was rapidly closed and a 5 s scrambled -slightly over threshold-foot-shock (0,5 mA) was delivered to the grid floor. Immediately after, the rats were returned to its home cage with food and water *ad libitum*. Twenty four hours later, a test session was carried out in a similar way, though without shock. Latencies to enter the dark compartment in both training and test sessions were recorded. For each rat (test-training), latencies difference was calculated as paired data to allow comparison within groups. Animals reached the learning criteria when latencies to enter the dark compartment were significantly higher during the test than during the training session, and the latencies difference was significantly higher than zero.

### Tail-Flick

This test was used to evaluate sensitivity and response to a painful stimulus (D'Amour and Smith, 1941). Briefly, to habituate the animal to the procedure, it was kindly suspended by hand and its tail was introduced three times (10 min apart) into a thermostatic water bath maintained at 20 ± 1°C, until tail withdrawal or up to 20 s. Then, 20 min later the tail was introduced into another bath at 51± 1°C. The latency to withdraw the tail from the bath was recorded.

### Novel Object LT-Recognition Task (NOR)

We used an adapted protocol with a 24 h-retention interval to test LTM. Four-month-old Tg+/– (N_Tg+/−_ = 19) and wt (N_wt_ = 16) rats were individually left to freely explore an OF in a 10 min session each day, along 3 consecutive days, for habituation to the environment. On the 4th day, two identical objects (A-A′) were added close to adjacent corners of the OF and then, each rat was left to re-explore the OF with the two objects for 5 min (training session or object exposure phase) (scheme in **Figure 3A**). The latency to start exploring an object and the time a rat spent exploring each object was recorded (and total object exploration was calculated afterwards). After object recognition (OR) training, rats were placed back into their home cages. On the 5th day (24 h after OR training), the rats were split into two groups containing a similar number of wt and Tg+/– rats. In the first group (control group, N_wt_ = 8, N_Tg+/−_ = 9), each rat was exposed for 5 min to the same familiar objects (A-A′). In the second group (test group, N_wt_ = 8, N_Tg+/−_ = 10), each rat was exposed for 5 min to a familiar and a novel object (A-B) (test session of NOR), in the same OF (scheme in **Figure 3A**).

Objects were similar in texture, color and size (≈10 cm high), but with distinctive shapes (i.e., pyramids vs. hemispheres). The different objects and their positions into the OF were counterbalanced across assays and behavioral trials. Discrimination Index (DI) was calculated as follows:

$$\begin{array}{l} Discrimination\;Index\;\left(DI\right)\\ =\frac{\left(time\;exploring\;object\;A^{\prime }\;or\;B\;-\;time\;exploring\;object\;A\right)}{\left(time\;exploring\;object\;A^{\prime }\;or\;B\;+\;time\;exploring\;object\;A\right)}. \end{array}$$

### Social Approach (SA)

A slightly modified version of the three chambered task for social approaching was used to assess social interaction and related memory (Moy et al., 2004; Yang et al., 2011) in naïve 4-month-old Tg (N_Tg+/−_ = 12, N_Tg+/+_ = 6) and wt (N_wt_ = 10) male rats. Briefly, 1 h before the task, an experimental rat (Erat) was kept alone in a separate cage, isolated from their littermates. Then, this Erat was left to freely explore an OF for 5 min (**Figures 4A**, **5A**, step 1) and was returned to the isolation cage. Two small cages were introduced in adjacent corners of the same OF and 10 min later, the Erat was left to explore the OF with the two empty cages for 5 min (training session, **Figures 4A**, **5A**, step 2), and it was again returned to the isolation cage. The time the Erat spent exploring each empty cage was recorded. Thereafter, an unknown novel rat (Nrat, of the same sex and age than the Erat) was placed inside one of the small cages, and an unknown object (of similar size and color as the Nrat) was placed inside the other cage. The 1st test session was carried out 10 min after training: The Erat was left in to the OF to explore for 5 min the arena with the Nrat in one cage and the object in the other (**Figures 4A**, **5A**, step 3). The time the Erat spent exploring the Nrat and the object was recorded. Once again, the Erat was returned to its home cage. In a 2nd test session, the Erat was left to explore the same arena, with the already known Nrat in one cage and a novel N'rat in the other (**Figure 4A**, step 4). This was conducted to test the capacity of an Erat to recognize and discriminate an already familiar congener from a novel one (it involves novelty, memory formation and active retrieval). The location of Nrat, object and N'rat were counterbalanced across assays. The time intervals an Erat spent exploring either N or N'rat were recorded and compared as explained in the Statistical Analysis section.

The mathematic definition of the Preference Index (PI) was always the same, although it was calculated from the exploration time data in each session as follows:

$$\begin{array}{l} PI\;for\;training\;session\\ =\frac{\left(time\;exploring\;cage\;A\;-\;time\;exploring\;cage\;B\right)}{\left(time\;exploring\;cage\;A\;+\;time\;exploring\;cage\;B\right)};\\ PI\;for\;1st\;test\;session\\ =\frac{\left(time\;exploring\;Nrat\;-\;time\;exploring\;object\right)}{\left(time\;exploring\;Nrat\;+\;time\;exploring\;object\right)};\\ PI\;for\;2nd\;test\;session\\ =\frac{\left(time\;exploring\;N^{\prime }rat\;-\;time\;exploring\;Nrat\right)}{\left(time\;exploring\;N^{\prime }rat\;+\;time\;exploring\;Nrat\right)}. \end{array}$$

### Statistical Analysis

For the OF and IA tasks (and Tail Flick), an upper time limit was specified for each test session. As consequence, both the number of crossings, test latencies and latencies differences did not follow a Gaussian distribution (D'Agostino & Pearson omnibus normality test) and these data were analyzed using non-parametric statistics (Kruskal Wallis Test or Mann Whitney) and expressed as medians with interquartile ranges (P25/P75).

To assess whether animals explored the new objects in the NOR task significantly more than what would be expected by chance (50%), Student's *t*-Test was carried out. The same test was used to uncover whether the animals explored the cage, the unknown novel rat (Nrat) or the new rat (N'rat) significantly more than what would be expected by chance, in the social approaching task (SA). All values are represented as mean ± SEM. Discrimination Index (DI) and Preference Index (PI) analysis were accomplished by using One-way ANOVA with Dunnett *post-hoc* test.

Differences between genotypes in qRT-PCR, ELISA, and Western blot analyses were assessed using a two-tailed, unpaired Student's *t*-test. Data are expressed as mean ± SEM. Significance was set at *p* < 0.05.

All data analysis was performed using the GraphPad Prism 6.01 program (GraphPad Software, Inc., San Diego, CA, USA).

### Brain Tissue Collection

Separate cohorts of 3-, 4-, and 6-month-old McGill Tg +/– rats and their wt littermates (3 mo: N_wt_ = 7, N_Tg+/−_ = 5; 4 mo: N_wt_ = 6, NT_g+/−_ = 5; 6 mo: N_wt_ = 6, N_Tg+/−_ = 5) were processed for brain tissue collection. As these rats were not subjected to behavioral tasks, subsequent analyzes reflect baseline protein and transcript levels. Rats were anesthetized with Equithesin (3 ml/kg ip) and perfused transcardially with ice-cold saline buffer. Their brains were removed, one hemisphere was kept at −80°C for molecular and biochemical analyses while the other hemisphere was post-fixed for 24 h in 4% paraformaldehyde, then saturated in 30% sucrose and processed for immunohistochemistry.

### RNA Extraction and Expression of Synaptic Plasticity-Associated Genes

Total RNA was extracted from hippocampi of 3-month-old (N_wt_ = 7; N_Tg+/−_ = 4), 4-month-old (N_wt_ = 4; N_Tg+/−_ = 4) and 6-month-old male rats (N_wt_ = 6; N_Tg+/−_ = 5) using the RNeasy Mini Kit (Qiagen) following manufacturer instructions, including an on-column DNase digestion step to remove residual DNA. Total RNA was then converted to cDNA using the iScript cDNA synthesis kit (Bio-Rad Laboratories, USA). The expression of synaptic genes was examined in triplicate using the primers listed in Table 1, the SsoAdvanced Universal SYBR Green Supermix (Bio-Rad) and the CFX Connect real-time PCR detection system and software (Bio-Rad). The relative expression of each transcript was calculated using the ΔΔCT method and standardized against the housekeeping genes 18S, GAPDH, HPRT and β-actin.

**Table 1:** Nucleotide sequences of primers used for qRT-PCR.

**Gene**	**5^**′**^-3^**′**^ Forward sequence**	**5^**′**^-3^**′**^ Reverse sequence**	**Amplicon (bp)**
*Grin1*	TAGGGCTATCACCTCCACCC	CTCCCTCTCAATAGCGCGTC	136
*Grin2a*	TGCGGGAACCCGCTAAACC	ATGGTCGCCACTTAGGGTCC	115
*Grin2b*	AACCCTCGTGGCCAGCA	CAGCTAGTCGGCTCTCTTGGTT	65
*Gria1*	TATGGAAGAGCAGACGTGGC	CACCTGGCTTGGACTTCTGT	136
*Arc*	GTTGACCGAAGTGTCCAAGC	CCGTCCAAGTTGTTCTCCAG	84
*Camk2β*	TTCCGACAGCACCAACACAA	CACTGATAGGGGTCCCTCGG	121
*c-fos*	CTCAGTTGCTAGCTGCAATCG	CCCCCTCCAGTTTCTCTGTT	113
*Egr1*	CTTGGATGGGAGGTCTTCAC	CGAATCGGCCTCTATTTCAA	145
*Dlg4* (PSD-95)	ACAACCAAGAAATACCGCTACCA	CCCCTCTGTTCCATTCACCTG	156
*Syn1*	GTGTTTGCCCAGATGGTTCG	TACCTTGACCTTGCCCATCC	165
18S	CATTCGAACGTCTGCCCTAT	GTTTCTCAGGCTCCCTCTCC	109
*Gapdh*	TGATGGGTGTGAACCACGAG	TCATGAGCCCTTCCACGATG	132
*Hrpt*	TTCCTCCTCAGACCGCTTTTC	CATCACTAATCACGACGCTGG	80
*β-Actin*	CTAAGGCCAACCGTGAAAAG	ACCAGAGGCATACAGGGACA	104

### Western Blotting

Hippocampi from wt or Tg+/– rats were homogenized in 100 mM NaCl, 0.2% Triton X-100, 1 mM EGTA, antiproteases cocktail (Sigma), 20 mM HEPES buffer (pH 7.4); and then incubated on ice for 30 min. Homogenates were cleared by centrifugation and the protein concentration in the supernatant was estimated using the BCA kit (Sigma). Samples were resuspended in Laemmli buffer and boiled at 100°C for 5 min. Protein samples were separated on a 10% SDS-PAGE gel and transferred to a polyvinylidenedifluoride (PVDF) membrane (Immobilon-P, Millipore). Blots were blocked with 3% non-fat milk-0.05% Tween-20 in Tris buffer saline (TTBS) and incubated with primary antibodies directed against: NR1 (rabbit polyclonal 1:1000, Sigma), NR2A (rabbit polyclonal, 1:1000 Chemicon), NR2B (rabbit polyclonal, 1:1000 Chemicon), PSD95 (mouse monoclonal 1:400, Abcam), CAMK2β (rabbit polyclonal, 1:1000 Abcam); and GAPDH (1:5000, Sigma). After wash-out, blots were incubated with the appropriate HRP-conjugated secondary antibody, anti-rabbit secondary antibody (1:2000; Amersham Biosciences, GE Healthcare, Piscataway, NJ, USA) or HRP-conjugated anti-mouse secondary antibody (1:5000; Sigma), developed in SuperSignal West Pico Chemiluminescent Substrate solution (Thermo Scientific, Waltham, MA, USA) and exposed to film (Agfa-Gevaert NV, Mortsel, Belgium). The intensity of bands corresponding to each NMDAR subunit was quantified with the gel analysis tool of Fiji software (NIH, Bethesda, MA, USA) (Schindelin et al., 2012) and normalized by the corresponding GAPDH band used as internal control.

### Immunohistochemistry

Free-floating immunostaining was done according to established protocols (Leon et al., 2010; Iulita et al., 2014b, 2017). Briefly, 40 μm coronal sections were incubated in 0.3% hydrogen peroxide solution for 30 min, washed three times in PBS-T (0.2 M phosphate-buffered saline, 0.2% Triton X-100) and blocked 1 h with 10% normal goat serum (NGS) in PBS-T. To examine amyloid pathology, the sections were incubated with a monoclonal antibody specific for the human Aβ peptide (McSA1 mouse monoclonal, 1:4000 in 5% NGS in PBS-T, MediMabs, Montreal) (Grant et al., 2000), overnight at 4°C. Sections were then incubated with a goat anti-mouse IgG secondary antibody (1:100 in PBS-T; MP Biochemicals, Canada) for 1 h, followed by 1 h incubation with a mouse anti-peroxidase monoclonal antibody (Semenenko et al., 1985) at 1:30, pre-incubated with horseradish peroxidase (5 μg/ml) (MAP kit, Medimabs, Canada). Immunohistochemical labeling was developed with 0.06% 3,3′-diaminobenzidine (Sigma-Aldrich, USA) and 0.02% hydrogen peroxide (Sigma-Aldrich, USA) in PBS. Sections were mounted on subbed slides and left to dry before being dehydrated in increasing concentrations of ethanol and cleared with xylene before they were coverslipped with Entellan mounting medium (EMD Millipore, USA). Images were taken by brightfield microscopy on an Axioplan 2 imaging microscope (Zeiss, Germany) equipped with an Axiocam 506 color camera (Zeiss) and running Zen Blue software (Zeiss).

### Quantification of Soluble Aβ42 Levels

Hippocampal tissue (10–15 mg) was homogeneized in eight volumes (v/w) cold TBS containing protease inhibitors, briefly sonicated and cleared by centrifugation at 15,000 × g at 4°C for 45 min. The levels of Aβ42 peptides were assessed in duplicate using the Amyloid beta 42 Human ELISA Kit (Invitrogen, Carlsbad, USA). Aβ42 concentrations were extrapolated from a standard curve of synthetic human Aβ42 (range: 15.6–1,000 pg/ml) and corrected for protein concentration, as determined by the Lowry assay, and are expressed as pg Aβ42 per mg protein.

## Results

### Open Field Exploration

**Figure 1:**
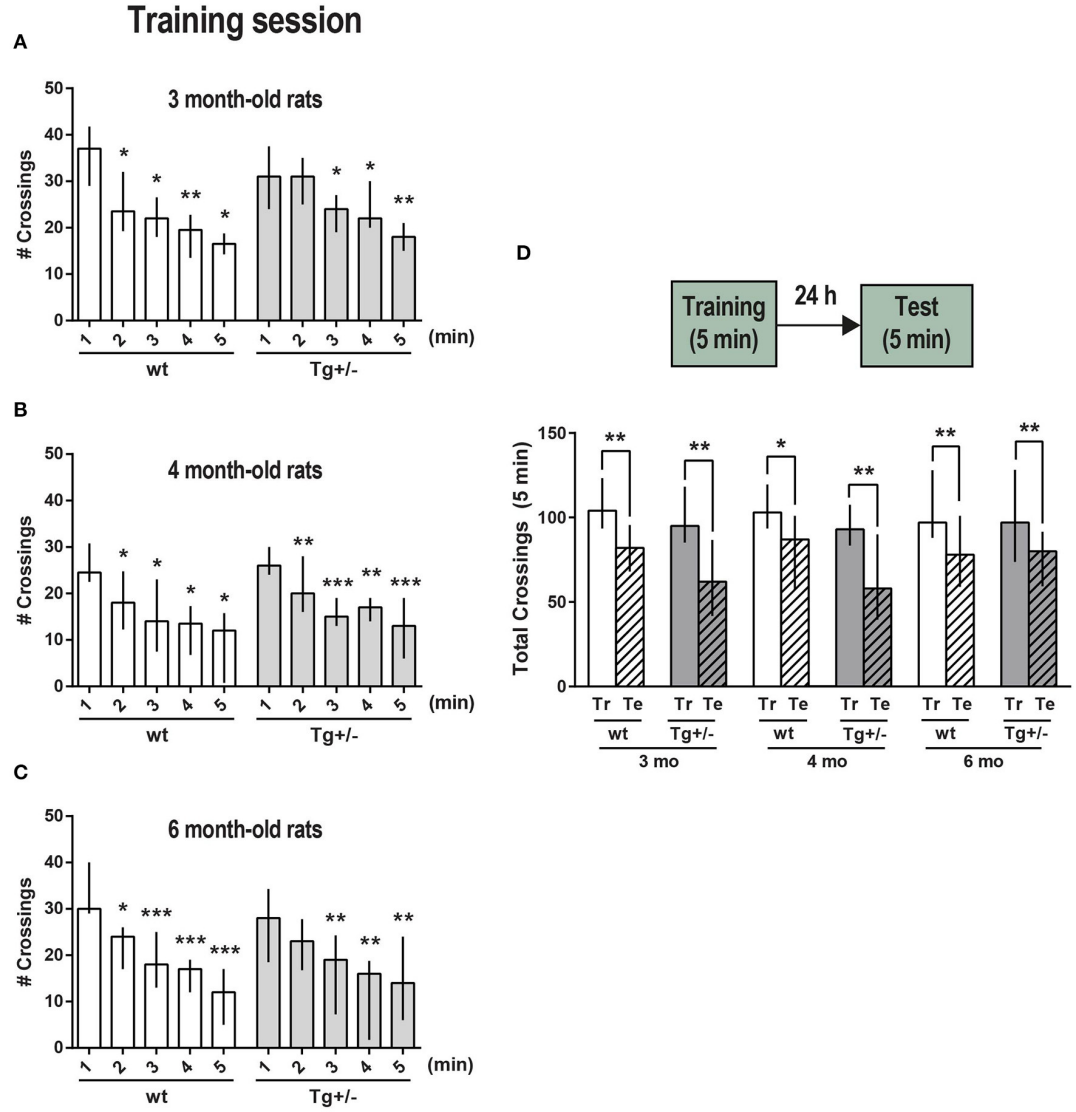
Exploration and habituation to an OF. McGill-R-Thy1-APP Tg+/– male rats (dark gray bars) and their wt littermates (white bars) were trained and tested for short-term (ST, intra-session) habituation to an OF at **(A)** 3, **(B)** 4, and **(C)** 6-months of age. Bar diagram shows median and interquartile ranges (25;75) for the number of crossings recorded during each minute of a 5 min session (training, Tr). Significant differences were assessed by Wilcoxon matched pairs signed rank test; *P < 0.05, **P < 0.01, ***P < 0.001 (3 mo: N_wt_ = 8, N_Tg+/−_ = 11; 4 mo: N_wt_ = 8, N_Tg+/−_ = 11; 6 mo: N_wt_ = 11, N_Tg+/−_ = 10). **(D)** Long-term (LT) habituation to an OF (LTM). Bar diagram shows median and interquartile ranges (25;75) for the total number of crossings recorded during a 5 min training session (Tr, plain bars) and during the test session (Te, stripped bars) which was performed 24 h later (schematic representation of the task steps on top). No significant differences were observed between any selected pair of training sessions amongst the wt or Tg+/– animals (Unmatched Kruskal-Wallis test with Dunn's multiple comparisons). Total crossings during test session were significantly lower than during training session for the three different ages, either for wt or Tg animals. Statistically significant differences were assessed by Wilcoxon matched pairs signed rank test; *P < 0.05, **P < 0.01 (3 mo: N_wt_ = 8, N_Tg+/−_ = 8; 4 mo: N_wt_ = 8, N_Tg+/−_ = 9; 6 mo: N_wt_ = 11, N_Tg+/−_ = 8).

Exploration of an open field (OF) is a simple behavioral paradigm, where habituation to the environment can be easily assessed. The spontaneous exploratory activity in an unique OF session has been previously reported in McGill-R-Thy1-APP transgenic (Tg) rats (Galeano et al., 2014). Here, the OF task was adapted to also test long-term (LT) habituation to the OF, as there was a 24 h interval between the initial exposure to the OF and the test session. Three-, 4- and 6-month-old Tg+/– rats and their wt littermates were left to freely explore the arena (Figure 1). At these time-points the incremental amyloid pathology is limited to the intraneuronal space of the hippocampus and cerebral cortex (Supplementary Figures 2A–C). Soluble Aβ42 peptides can be detected in the hippocampus using a well-established ELISA procedure (Supplementary Figure 2E), as previously reported (Do Carmo et al., 2016). Although soluble Aβ42 levels were not significantly different between 3- and 4-month-old Tg rats, there were significant differences for 3- and 4- compared to 6-month-old rats (Supplementary Figure 2E), denoting a progressive pathology.

During a 5 min session -named training session (Tr)-, the number of crossings from one square to another across the lines drawn on the floor was recorded minute by minute. This number of crossings per minute was compared with the first minute in the OF, being lower either in the second or third minute for Tg+/– rats, and in the second minute for wt rats, indicating that both were able to recognize and habituate to the environment, and denoting an active short-term memory (STM) of the environment at 3-, 4-, and 6-months of age (Figures 1A–C, respectively). To assess “vertical exploration,” the number of rearings was also recorded for 4- and 6-month-old rats. A significant decrease in the number of rearings for both Tg+/– rats and their wt littermates corroborated the proper habituation to the environment and the presence of STM (Supplementary Figures 3A,B). Each rat' position in the experimental arena was tracked in real-time. The overall trajectory and the distance traveled were assessed (Supplementary Figures 4A–C); the time spent in corners and periphery (edges) vs. center (Supplementary Figures 4D,E), resting time and grooming events were also quantified for 4- and 6- month-old rats along the 5 min session (Supplementary Figure 4F). The comparison between data for all groups showed that there were no significant differences between them regardless age and genotype (Supplementary Figures 4A–E). Both 4- and 6-month-old Tg and wt rats did not show freezing behavior in this first exposure to the OF. To assess long-term memory (LTM) of the environment and LT habituation, some rats were left to freely explore the same OF along 5 min (test session, Te), 24 h after the Tr session (Figure 1D). Total number of crossings as well as total number of rearings along this training session, were not significantly different between wt and Tg+/– rats of the same age, neither between rats of different ages, showing that the locomotor and exploratory activity was rather similar. Both Tg+/– and wt rats showed a significant decrease in the total number of crossings in the test session, indicating that there was LT habituation to the environment and that a LTM was consolidated and active at the three different ages. A significant drop-down in the total number of rearings 24 h after training, also accounts for LT habituation and for an active LTM (Supplementary Figure 3C).

### Inhibitory Avoidance

**Figure 2:**
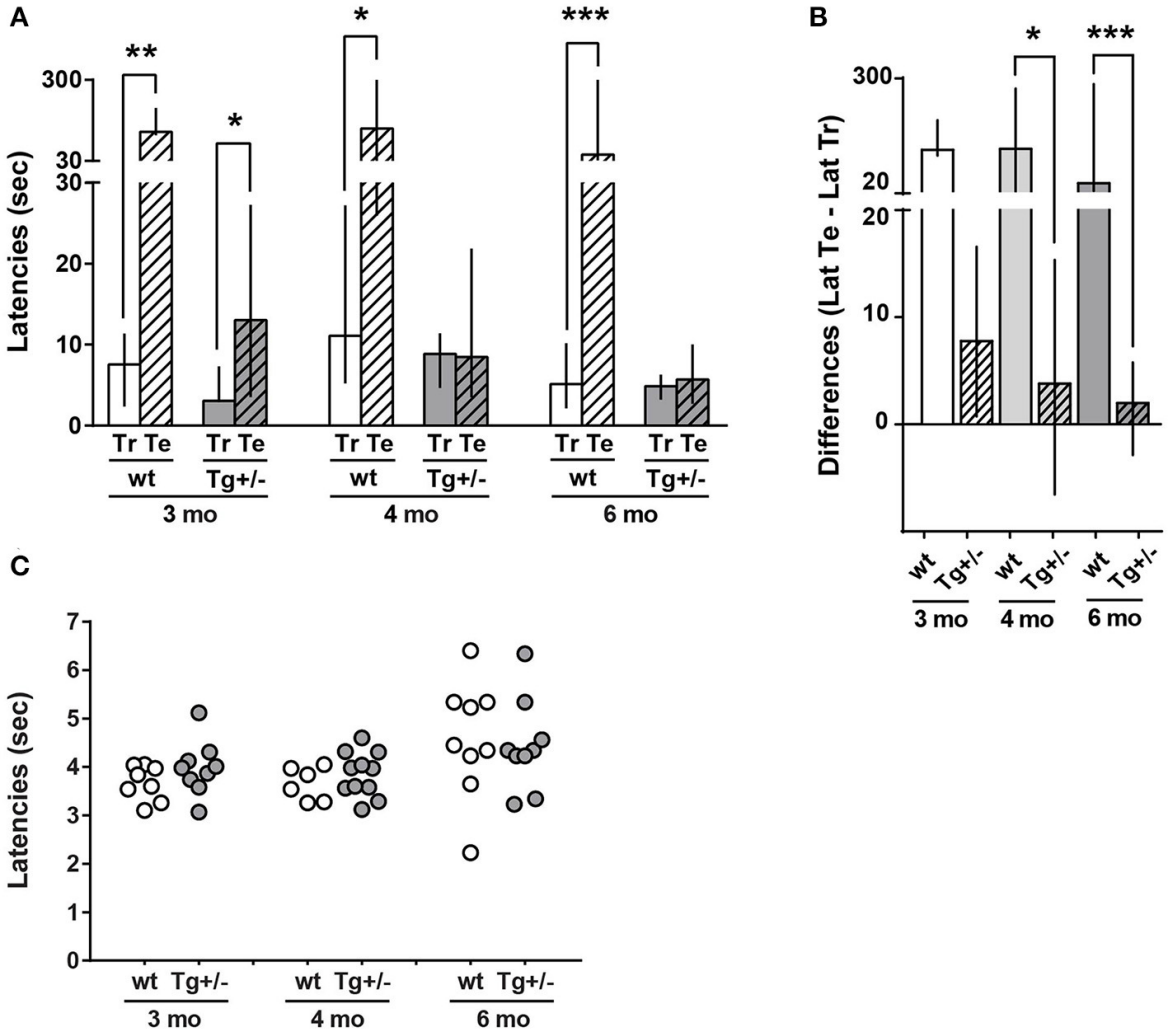
Long-term memory (LTM) of inhibitory avoidance (IA) in 3-, 4-, and 6-month-old McGill-R-Thy1-APP Tg+/– rats. **(A)** IA task: Training and Test Latencies. Tg+/– (dark gray bars) and wt littermates (white bars) were trained in a step through IA to a mild foot-shock (0.5 mA) and tested 24 h later. Latencies to enter the dark compartment (from a lighted one) were recorded. The bar diagram represents median of latencies and interquartile ranges (25;75). Test latencies (Te, stripped bars) to enter the dark compartment were significantly higher than training latencies (Tr, plain bars) for 3, 4, and 6-month-old wt rats. However, only 3-month-old Tg+/– rats showed a significant difference between Te and Tr latencies. Neither 4- nor 6-month-old Tg rats showed differences between Te and Tr latencies. Significant differences were assessed by Wilcoxon matched pairs signed rank test; *P < 0.05, **P < 0.01, ***P < 0.001. **(B)**. IA task: (Te – Tr) Latencies Difference. Bar diagram represents median of (Te-Tr) difference of latencies, with interquartile ranges (25;75) for the same groups as in A. Differences were significantly lower in Tg+/– (stripped bars) than in wt (plain bars) rats of 4 (light gray) and 6 (dark gray) months, whilst there were no statistically significant differences among the difference of latencies by genotype in 3-month-old rats (Unmatched Kruskal-Wallis test with Dunn's multiple comparisons. *P < 0.05, ***P < 0.001). Tt-Tr latency difference of 3-month-old Tg+/– rats was significantly different form zero, while latency differences for 4- and 6-month-old Tg+/– rats were not different from zero. Unmatched Kruskal-Wallis test with Dunn's multiple comparisons. *P < 0.05) (3 mo: N_wt_ = 8, N_Tg+/−_ = 7; 4 mo: N_wt_ = 7, N_Tg+/−_ = 11; 6 mo: N_wt_ = 11, N_Tg+/−_ = 13). **(C)** Tail-Flick latency. Tail withdrawal latency from a hot (51 ± 1°C) water bath is represented in the scatter plot. No significant differences were observed between any selected pair of animals (Tg or wt) of a given age (Unpaired Mann-Whitney test).

The inhibitory avoidance (IA) of a foot-shock is a classic associative task involving both aversive and spatial components, currently used to evaluate learning and memory acquisition in one short session. Three, 4- and 6-month-old Tg+/– male rats as well as their wt littermates were individually subjected to a training session. A test session was performed 24 h after the training session. In both sessions, latency to go across a sliding door and enter a dark –preferred- compartment, was recorded (Figure 2A). During the training session, none of the rats evidenced freezing behavior in the enlighten compartment and the latency to enter the dark compartment did not differ significantly across genotypes and ages. As expected, in wt rats, latency to enter the dark compartment in the test session was significantly higher than in the training session, independently of age, showing that wt rats remembered how to avoid the aversive stimulus by inhibiting to run away to the dark side (Figure 2A); hence, a LTM of IA has been formed and was active. In contrast, only 3-month-old Tg+/– rats successfully completed the task as their latency to enter the dark compartment in the test session was significantly higher than latency in the training session, while 4- and 6-month-old Tg+- rats showed similar latency values in both sessions (Figure 2A). The differences between test and training latencies (paired values) are shown by age and genotype in Figure 2B. Dunn's multiple comparisons *post-hoc* test of all groups' latency differences (Unmatched Kruskal Wallis test) showed that there are statistically significant differences among wt and Tg rats at 4- and 6-months though not at 3-months. These results indicate that 3-month-old Tg+/– male rats learned and remembered the task similarly to wt rats, whereas 4 and 6-month-old Tg+/– male rats did not, denoting serious deficits in IA LTM. However, it should be noted that the latency in the test session was significantly lower in 3-months old Tg+/– rats compared with wt rats, suggesting that although these Tg rats achieved the criteria for the task, they would suffer some mild deficit in IA LTM performance. In addition, when comparing latencies differences among Tg rats, those of 6-month-old resulted significantly lower compared to those of 3-month-old (*p* < 0.05; Mann Whitney test); neither 3- vs. 4-month-old nor 4- vs. 6-month-old were significantly different. The results suggest that there was a progressive impairment among these relatively close ages.

Since McGill-R-Thy1-APP Tg+/– rats impaired performance in IA task could be explained by either a decrease or lack of sensitivity, a tail-flick assay was performed on the same animals (Figure 2C). The analysis revealed that both Tg and wt rats showed similar latencies for tail withdrawal from a hot water bath at 51°C±1°C, at the three different ages, indicating that Tg+/– male rats were able to sense an aversive stimulus in a similar way as wt animals do. Therefore, this strongly suggests that 4- and 6-month-old Tg+/– rats did not form an appropriate LTM of the IA task.

### Novel Object Recognition (NOR)

**Figure 3:**
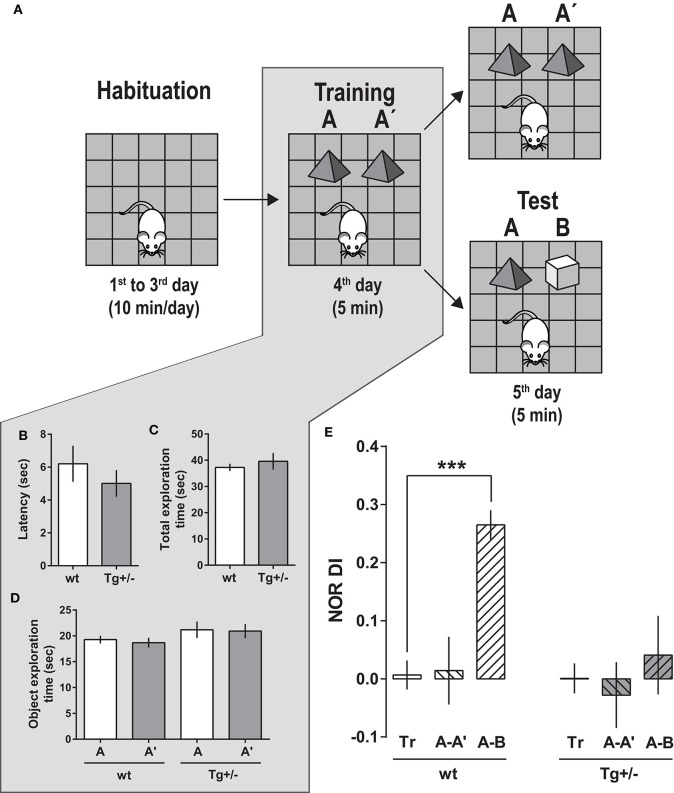
Novel object recognition (NOR) task performance by 4-month-old McGill-R-Thy1-APP Tg+/– rats **(A)** Schematic representation of the task steps. Habituation: Rats were left to freely explore an OF for habituation to the environment (10 min/session, along 3 days). Training (Tr): On the 4th day, each rat was left to re-explore the OF in the presence of two identical objects (A–A′), for 5 min. Test (Te): On the 5th day, trained rats were split in two groups: Rats from one group were exposed for 5 min to the same two objects (A–A′) (as internal control at 24 h), while rats from the other group, to a familiar and a novel object (A, B). **(B)** Training. Latency to start exploring an object. Bars represent latencies as mean ± SEM for each group. There were no significant differences between Tg+/– (dark gray bars, N_Tg+/−_ = 19) and wt (white bars, N_wt_ = 16) rats (Student's *t*-Test). **(C)** Training. Total time spent exploring objects (A+A′) by Tg/+- and wt rats during the 5 min Tr session. No significant differences were found between groups (Student's *t*-Test). **(D)** Training. Time spent exploring each object (A or A′). There were no significant differences when comparing the time spent exploring A and A′ (Paired-*t*-test). **(E)** Test. On the 5th day, when tested in the presence of a novel (B) and a familiar (A) object (A-B, striped-bars), wt rats showed a significantly higher *Discrimination Index* in the Test session: *[DI*_*Te*_ *(A-B)* = *(time exploring B - time exploring A)/(time exploring B* + *time exploring A*)*]*, compared to the *Discrimination Index* of Training *[DI*_*Tr*_ *(A-A*′*)* = *(time exploring A*′ *- time exploring A)/(time exploring A*′ + *time exploring A)]* (plain bars) or against zero. At variance, DI_Te_ (A-B) for Tg+/– rats was similar to DI_Tr_ (A-A′)—and it was not significantly different from zero. The two other groups of Tg+/– and wt rats were tested with same objects (A-A′) used in the Tr session, as an internal control after 24 h of Tr. In this case, the DI_Te_ (A-A′) for Tg+/– as well as that for wt rats were similar to the respective DI_Tr_. One-way ANOVA, Dunnett *post-hoc* test; ***P < 0.001 [N_wt_ Te (A-A′) = 8; N_wt_ Te (A-B) = 8; N_Tg+/−_ Te (A-A′) = 9; N_Tg+/−_ Te (A-B) = 10].

Since 4-month-old Tg+/– rats showed a LTM impairment in the IA task, we then tested their capacity to discriminate a novel object from a familiar one using a LT-adapted novel object recognition (NOR) task (Figure 3A). During the training session (exposure phase), rats were presented with two identical objects A and A′ into the familiar OF. Tg+/– and wt rats showed similar latencies to start exploring the objects (Figure 3B). In addition, Tg+/– and wt rats showed a similar total exploration time of both objects (Figure 3C), as well as similar amount of time exploring objects A and A′ (Figure 3D) in this first session.

Twenty-four hours later, half of the animals were presented again with the same two identical objects (A-A′ test session) (control group), and the other half, with a familiar object (A) and a novel object (B) (A-B test session) (Figures 3A,E). Tg+/– and wt rats exposed to same A-A′ objects used in the training session did not show any significant difference in the exploration parameters for both objects, neither in the calculated discrimination index (DI) (Figure 3E). However, during the (A-B) test session, wt animals spent more time exploring the novel object B than the familiar A. In consequence, the DI for the test session was significantly higher than the DI for training and significantly different from zero, indicating that wt animals remembered, recognized and discriminated the familiar from the novel object (Figure 3E). In contrast, Tg+/– rats spent a similar amount of time exploring the novel object B and the familiar object A; the DI of the test session was not significantly different from the training DI, neither different from zero.

Our results show that after a retention interval of 24 h, Tg+/– rats were unable to discriminate a familiar from a novel object, indicating that 4-month-old animals have an impairment in LTM of objects and in using this memory for object discrimination.

### Social Approach

**Figure 4:**
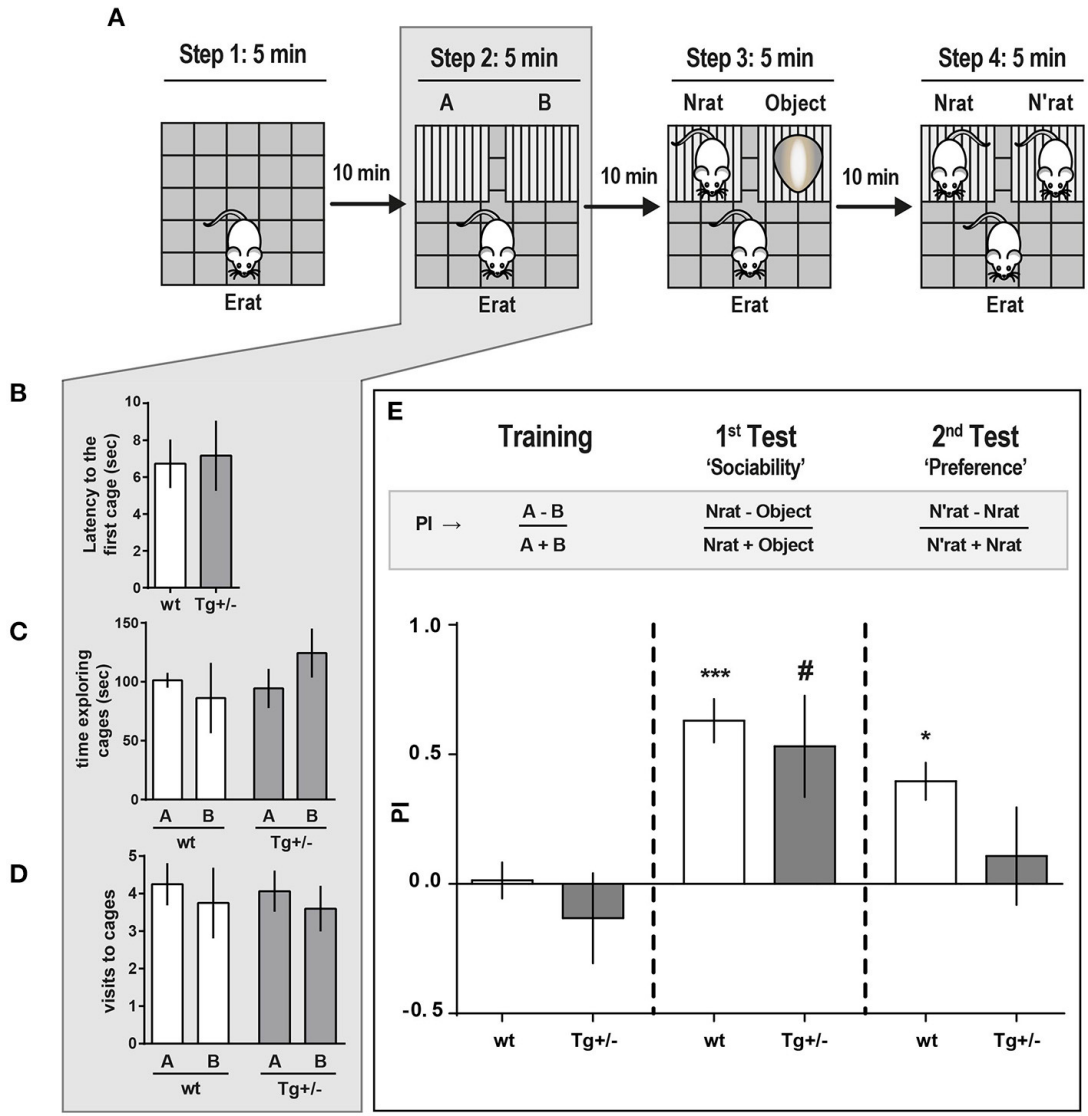
Social approach behavior of McGill-R-Thy1-APP Tg+/– rats. **(A)** Schematic representation of the task steps. Tg+/– 4-month-old rats and their wt littermates were tested in an adapted social interaction-three chambered task. Step 1. Habituation: Erat was left to freely explore an OF for 5 min. Step 2. “Training”: Exploration of the OF with two empty cages added. Erat was left to explore the same OF now containing cages A and B, for 5 min, and the time Erat spent exploring each cage was recorded. Step 3. “Sociability”: An unknown novel rat (Nrat, same sex, and age as Erat) was placed inside one cage, and an unknown object (similar size and color as the Nrat) was placed in the other cage. The time Erat spent exploring either the Nrat or the object over 5 min was recorded. Step 4. “Preference for Social Novelty”: 10 min after Step 3, Erat was left to explore the OF with the already known Nrat in one cage and a novel N'rat (same sex and age of Nrat), in the other. Time Erat spent exploring either N or N'rat was recorded. **(B)** Latency to start exploring empty cages (A or B) (Step 2 of scheme in A) was similar for Tg+/– (dark gray bars, N_Tg+/−_ = 12) and wt (white bars, N_wt_ = 10) rats (bars represent mean latency ± SEM; Student *t*-test). **(C)** Time Erat spent exploring A or B empty cages. There were no significant differences between Tg+/– and wt rats (bars represent mean exploration time ± SEM; Paired *t*-test). **(D)** Number of visits either to A or B cages were not significantly different regardless the genotype (bars represent mean number of visits ± SEM; Paired *t*-test). **(E)** Preference Index (PI) for the three phases of the task: “Training” (control for location/cage (lack of) preference), “Sociability” and “Social Novelty.” “Training” (Step 2 of scheme in A). *PI* = *A-B/A*+*B* was not significantly different from zero for both genotypes, indicating that wt and Tg+/– Erats spent similar time exploring each cage, without any location/cage preference. 1st Test: “Sociability” (Step 3 of scheme in A). Both Tg+/– and wt Erats spent significantly more time exploring the Nrat than the object ('Sociability: *PI*_*Nrat*−0*bject*_ = *Nrat-Object/Nrat*+*Object* > 0). 2nd Test: “Preference for Social Novelty” (Step 4 in A). Wt rats explored significantly longer time the N'rat than the Nrat (“Preference,” ${\mathrm{PI}}_{wt\text{\_}N^{\prime }rat-Nrat}$ = *N'rat-Nrat/N'rat*+*Nrat* > 0), while Tg+/– rats spent similar time exploring the N'rat and the Nrat (“Preference,” ${\mathrm{PI}}_{Tg+/-\text{\_}N^{\prime }rat-Nrat}$ ≈ 0). ***P < 0.001, *P < 0.05, #*P* < 0.05; One-way ANOVA, Dunnett *post-hoc* test.

To characterize social behavior in 4-month-old Tg+/– rats, we then applied a modified three-chambered social approach paradigm. Ten minutes after habituation to the OF (Figure 4A, step 1), either a Tg+/– or wt experimental rat (Erat) was left to freely explore the OF, now with the addition of two small empty cages fixed to adjacent corners of the arena (training session, Figure 4A, step 2). There were no significant differences in the latency to start exploring a cage (Figure 4B), neither in the time spent exploring each cage, nor in the number of times visiting each cage (Figures 4C,D, respectively). For the training session, the $Preference\;Index\;\left(PI\right)=\frac{A-B}{A+B}$ (where A and B represent the time each rat spent exploring the respective cage) was not significantly different from zero, indicating no favored cage/side (Figure 4E, Training). The lack of preference for a particular cage/side during training is a mandatory requirement for the following steps. To assess “sociability,” defined as the Erat tendency to spend time with another rat, compared to time spent exploring an unanimated object, the following session (1st test, Figure 4A, step 3) was carried out 10 min later. For this, either an unknown rat (Nrat) or an object (similar size and color as the Nrat) was introduced into each cage. Both Tg+/– rats and wt littermates explored the Nrat for significantly longer time than the object. Hence, the sociability behavior was similar –and “normal'- for both groups of rats as they were able to discriminate and prefer the Nrat over the object (In this 1st test, $PI=\frac{\left(time\;exploring\;Nrat\;-\;time\;exploring\;object\right)}{\left(time\;exploring\;Nrat\;+\;time\;exploring\;object\right)}$; PI_Tg+/_: 0.63 ± 0.08; PI_wt_: 0.53 ± 0.18, respectively) (Figure 4E, “Sociability”). In the following session, performed 10 min later, the experimental subject (Erat) was exposed to the already familiar Nrat in one cage and a new N'rat, in the other (2nd test, Figure 4A, step 4). Although the mathematic definition for PI is the same, here it accounts for “social novelty preference,” which could be defined as a propensity to spend more time with a novel congener than with a familiar one. ($PI\;for\;2nd\;test\;session=\frac{\left(time\;exploring\;N^{\prime }rat\;-\;time\;exploring\;Nrat\right)}{\left(time\;exploring\;N^{\prime }rat\;+\;time\;exploring\;Nrat\right)}$). Wild type rats spent significantly more time exploring the novel N'rat compared with the time exploring the familiar Nrat (${\text{PI}}_{\text{wt}{\text{N}}^{\text{′}}-\text{N}}$: 0.40 ± 0.07). Conversely, there was no difference between the time Tg+/– rats spent exploring the Nrat and the N'rat, which indicates that they seemed to be indifferent to social novelty and strongly suggests that they did not recognize/discriminate an “already known” Nrat from a new unknown N'rat (${\text{PI}}_{\text{Tg}+/-{\text{N}}^{\text{′}}-\text{N}}$: 0.10 ± 0.18) (Figure 4E, “Preference”).

So far, our findings indicate that, in addition to the deficits in STM previously reported by other groups, 4-month-old Tg+/– rats display an impaired LTM and significant alterations in social behavior, in the absence of amyloid plaques.

**Figure 5:**
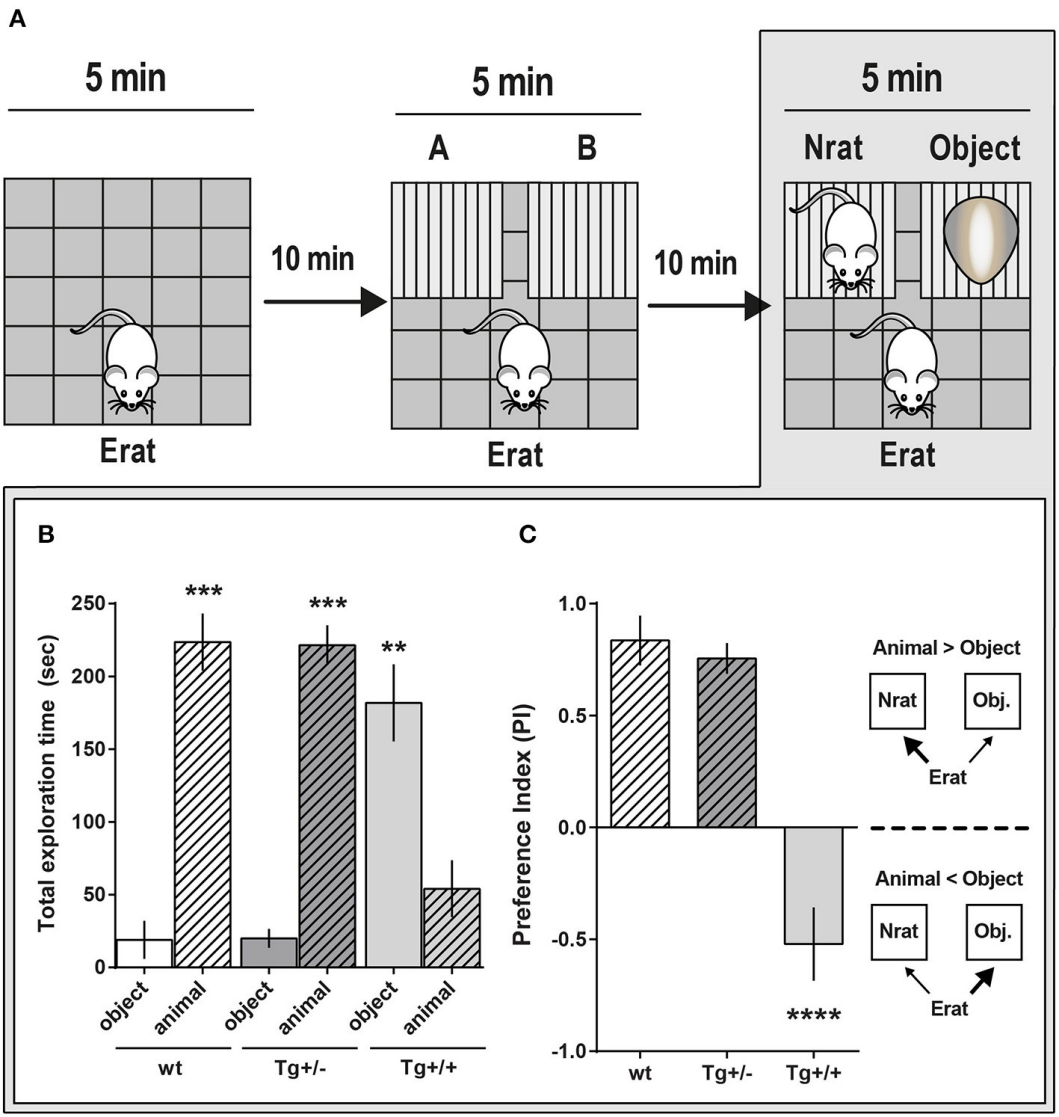
Social approach comparative behavior of McGill-R-Thy1-APP Homozygous Tg+/+ and Heterozygous Tg+/– rats. **(A)** Schematic representation of the task steps. 4-month-old Tg+/+, Tg+/– rats and their wt littermates were tested in an adapted version of the three-chambered task. Step 1: OF habituation. Step 2: Control for location/cages (lack of) preference. Erat explores the OF with A and B empty cages. Step 3: “Sociability” assessment. Erat explores the OF with a Nrat (novel rat, same sex, and age) in one cage, and an object (similar size and color than Nrat) in the other cage. **(B)** “Sociability”: Total exploration time of targets (Nrat or Object) during the 5 min session (Step 3 in A). Bar diagram represents the time (mean ± SEM) an Erat spent exploring an unknown Nrat, or an unknown object (O). Tg+/– animals (dark gray bars, N_Tg+/−_ = 13), Tg+/+ animals (light gray bars, N_Tg+/+_ = 6), wt littermates (white bars, N_wt_ = 6). Object (O): stripped bars; Novel animal (Nrat): plain bars. Tg+/– and wt rats spent more time exploring a Nrat than the object; while Tg+/+ rats spent significantly more time exploring the object (***P < 0.001, **P < 0.01, Paired *t*-test). **(C)** Sociability Preference Index (PI = Nrat–O/Nrat+O) was calculated using the data shown in *B*. During the social task, wt (white bars) and Tg+/– (dark gray bars) Erats spent more time exploring the Nrat than the object (PI_N−0_ > 0; plain bars), while Tg+/+ Erats spent more time exploring the object than the Nrat (PI_N−0_ < 0; stripped bars). ****P < 0.0001, One-way ANOVA, Dunnett *post- hoc* test.

We then examined whether 4-month-old Tg+/+ had more pronounced deficits in the three-chambered social approaching task compared to their Tg+/– and their wt littermates (Figure 5A). In an independent experiment we corroborated the result reported above in the 1st test (Figure 4E): When a novel Nrat was introduced into one of the small cages and an object into the other, both Tg+/– and wt experimental rats (Erats) spent more time exploring the Nrat than the object (Figures 5B,C). However, and unexpectedly, Tg+/+ rats spent significantly more time exploring the object than the Nrat (PI_Tg+/+_: −0.52 ± 0.18) (Figures 5B,C). This strongly suggests that the preference for social interaction is seriously disrupted in 4-month-old Tg+/+ homozygous male rats.

### Expression of Synaptic Plasticity-Associated Genes

**Figure 6:**
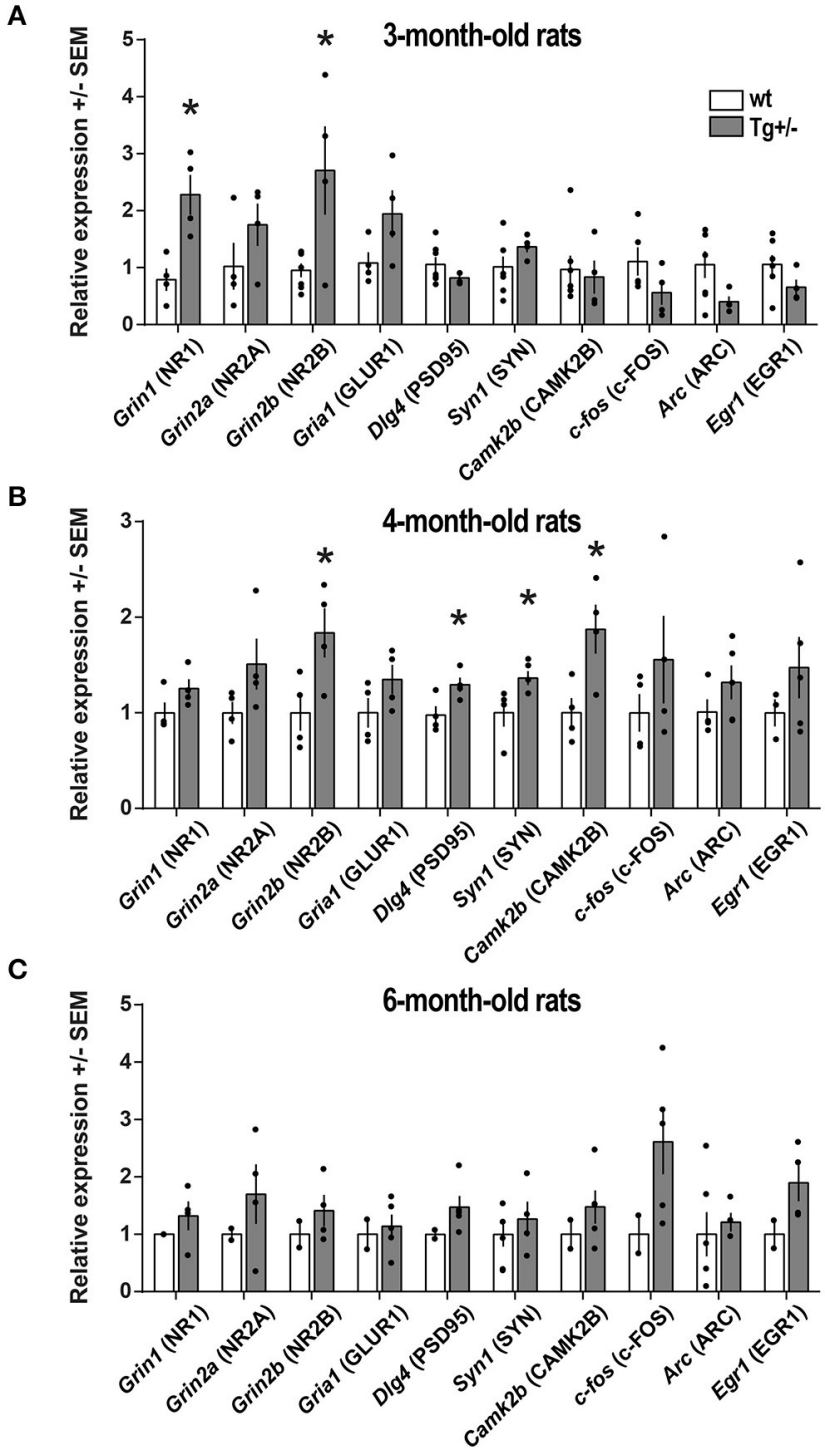
Expression of synaptic plasticity-associated genes. Total RNA was extracted from the hippocampus of **(A)** 3-month-old (N_wt_ = 7; N_Tg+/−_ = 4), **(B)** 4-month-old (N_wt_ = 4; N_Tg+/−_ = 4) and **(C)** 6-month-old (N_wt_ = 6; N_Tg+/−_ = 5) McGill-R-Thy1-APP Tg+/– male rats and their wt littermates. The relative expression of each transcript was calculated using the ΔΔCT method and standardized against the housekeeping genes 18S, GAPDH, HPRT, and β-actin. The name of the corresponding protein product is also indicated. Statistically significant differences were assessed by a two-tailed, unpaired *t*-test; *P < 0.05.

We then examined whether the cognitive deficits observed in Tg+/– at 4-months of age would be accompanied by an altered expression of genes related to synaptic plasticity. In the hippocampus of 4-month-old male Tg+/– rats (Figure 6B)—though neither of 3- nor of 6-month-old (Figures 6A,C) -, the transcript levels of *Dlg4, CamkII*β, and *Syn1* were significantly higher compared to their wt littermates (Figure 6). However, the levels of *c-fos, Egr1*, and *Arc* transcripts were not significantly different among both genotypes at the three ages.

We also examined transcript levels of NMDA receptor channel subunits *Grin1, Grin2a*, and *Grin2b*, largely known to be involved in synaptic plasticity and long-term potentiation (Gardoni et al., 2009; Baez et al., 2018). In 4-month-old male Tg+/– rats, the level of *Grin2b* transcripts was significantly higher compared to their wt littermates (Figure 6B). In agreement with transcript levels, PSD-95 and CAMK2β protein levels were also elevated in 4-month-old male Tg+/– rats compared to wt littermates (Supplementary Figure 5). Although it appeared to be some trend to an increase in NR2B protein level, it was not significantly different in Tg+/– and wt rats (p≈0,2). Interestingly, 3-month-old Tg+/– rats displayed elevated levels of *Grin1* and *Grin2b* while not statistically significant differences were detected in expression levels for the other genes examined in Tg+/– and wt rats (Figure 6A). In addition, at 6-months of age, all genes examined displayed similar expression levels in Tg+/– and wt rats (Figure 6C).

## Discussion

Alzheimer's disease (AD) is a complex neurodegenerative condition characterized by the progressive deterioration of memory and other cognitive functions, for which there is no cure. McGill-R-Thy1-APP Tg rats represent a valuable tool to better understand the amyloid-driven AD-like processes (Leon et al., 2010; Iulita et al., 2014a) and to evaluate the efficacy of experimental drug treatments in delaying or halting cognitive decline (Pimentel et al., 2015). Since drug interventions are more likely of being successful if applied early in the AD continuum in the absence of neuronal death, we thus deem of importance to further characterize the early behavioral impairments of these Tg rats, prior to the deposition of amyloid plaques.

The presence of learning and memory deficits in McGill-R-Thy1-APP rats is well-documented in the literature (Leon et al., 2010; Galeano et al., 2014; Iulita et al., 2014a). However, social interaction was not previously characterized in this model and long-term memory (LTM) impairments, although well-characterized in AD, have attracted far less attention. In the present study, we showed that, in addition to the memory deficits previously reported, 4-month-old Tg+/– male rats display an impaired LTM for inhibitory avoidance and object discrimination, as well as significant alterations in social behavior. These deficits in LTM seem independent of deficits in spatial memory for navigation as Leon et al. (2010) reported that, prior to the deposition of plaques, 3-month-old Tg+/– rats (females + males altogether) performed as well as wt rats in the MWM; however, Tg+/+ rats presented deficits in the MWM, in spite of intensive learning along 5 days. In addition, although 13-month-old Tg+/– rats showed a slower learning curve in the MWM compared to wt rats, there were not differences in memory retrieval in the fifth day; hence, spatial memory for navigation would be preserved in these heterozygous rats (Leon et al., 2010).

More specifically, our findings demonstrate that although LTM of habituation to the environment (OF) was preserved in 3-, 4-, and 6-month-old Tg+/– male rats, the last two showed severe memory impairments in other more complex and associative behavioral tasks. In the inhibitory avoidance (IA) to a mild foot-shock task, 3-month-old Tg+/– males were able to appropriately retrieve memory 24 hours later, staying for a longer period of time in the lighten compartment, instead of running to the “preferred” dark compartment. Hence, they formed an associative memory which was still active on the following day, though the corresponding test latencies were significantly lower than in wt rats. Since they did not show any significant difference in locomotion or exploratory behavior (as observed in the OF), this could be interpreted as the beginning of mild cognitive deficits; however, Tt-Tr latencies difference was significantly different from zero indicating that 3-month-old Tg+/– rats were able to remember, while latencies difference for 4- and 6-month-old Tg+/– rats was not significantly different from zero, denoting a severe impairment in the IA performance, which progressed with age.

These differences were not related to the capacity to sense the aversive stimulus as there were no significant differences among tail-flick latencies in 4-month-old Tg+/– rats compared to their wt littermates. Therefore, these results strongly suggest that in Tg+/– male rats associative LTM involving aversive and spatial cues is seriously impaired at 4-months of age. This deficit is likely due to alteration in consolidation and/or retrieval/activation of memory.

Our findings also show that 4-month-old Tg+/– male rats have deficits for LTM of objects and/or for objects discrimination since in the LTM-adapted NOR paradigm, these rats were unable to discriminate a familiar from a new object (or to prefer novelty as wt did). This result was surprising as it was previously shown that 3-month-old Tg+/– and Tg+/+ rats were able to achieve the learning criteria for a NOR task shortly after training; although there was a significant difference between performance of these two Tg genotype variants and that of their wt littermates (Iulita et al., 2014a). Furthermore, Pimentel et al., showed that 9-month-old Tg+/+ rats (females + males altogether though maintaining similar numbers) were able to learn to discriminate objects after a relatively short period (51 min after 1st familiarizing session), and to form a STM (Pimentel et al., 2015), precluding the possibility that performance could be affected either by some specific sensory modality alteration or by acquisition deficits. Interestingly, España et al. (2010) reported that APP_Ind_, APP_Sw,Ind_, and 3xTg-AD transgenic mice developed fear responses at 6-month—coinciding with accumulation of intracellular soluble Aβ monomers/oligomers-, when exposed to a bright light stimulus, consistent with reduced exploratory activity and increased anxiety. It was also suggested that 6- though not 3-month-old McGill Tg rats, developed some anxious-like behavior in the spontaneous exploration of an OF, as they spent less time in center than in periphery compared with wt littermates (Galeano et al., 2014). Although we could not preclude the possibility that some neophobic behavior could be affecting the interaction with a novel object and consequently the NOR performance, it was not evident in the short term-NOR task with de novel object (Iulita et al., 2014a), neither in 9-month-old rats (Pimentel et al., 2015), nor in our assays in the OF or in the IA task either.

Therefore, altogether our data and those in the literature strongly suggest that memory consolidation or its activation, for object discrimination and/or novelty preference, is impaired in 4-month-old Tg+/− male rats, impairing or impeding LTM formation and expression. In close agreement with these results, longitudinal studies using electrophysiological recordings in freely behaving Tg male rats revealed an age-dependent, relatively rapid-onset and persistent inhibition of (NMDA receptor-dependent) LTP, without baseline synaptic transmission (AMPA receptor-dependent) modification in the CA1 region of the hippocampus (Qi et al., 2014). A standard high-frequency stimulation −200 Hz− was unable to induce NMDA receptor dependent short- and long-term potentiation in 3.5-month-old Tg rats—in coincidence with the onset of cognitive decline at 3 (Tg+/+) and 4-month-old (Tg+/−) −, and that deficit in potentiation was shown to persist for another 2–3-months, when plaques would start to appear (Qi et al., 2014).

Moreover, given that rats are social animals, social approaching behavior should be relevant as part of their normal development. As far as we know, there was not previous report on social behavior in heterozygous rats and only one report in homozygous rats (Petrasek et al., 2018), where an alteration in social interaction was found. However, the paradigm used was different from the one applied in the present study and did not produce the expected observations in wt rats, resulting in inconclusive data on social memory. Herein, during the exposure to empty cages 4-month-old Tg+/–, Tg +/+, and wt rats behaved in a similar way, but their preference behavior differed greatly in the test session. Since Tg+/– animals were able to recognize and choose to approach and explore for longer a congener (the Nrat) than exploring an unanimated object, we can assume that there is not any evident alteration of specific sensory modalities -like olfaction and sight- in those animals that could affect their behavior. Therefore, Tg+/– rats deficit in discriminating an already known (N) from a new (N') rat would mainly be related to an impairment in cognitive functions, such as memory formation and/or expression. However, the fact that Tg+/+ rats explored for significantly longer time an object than a rat congener, showing a strikingly different behavior compared to Tg+/– rats, suggests a deep alteration in social interaction. Alternatively, as they do not prefer to interact with a congener (or with an unknown one?), it suggests that they might be unable to discriminate/recognize it. This observation is most likely independent of impairment in a specific sensory modality -e.g., sight or olfaction- as this would result in an absence of any preference, and thus, the exploration of the Nrat and the object would have been similar. These observations are puzzling and call for further studies. Nevertheless, we can speculate that this behavior might be related with the development of high anxiety levels, likely leading to increased fear in these rats, and hence, causing them to avoid interacting with an unknown animal. As cited above, España et al. (2010) reported that three different transgenic mice models of AD exposed to a bright light stimulus developed fear behavior at 6-months of age, consistent with reduced exploratory activity and increased anxiety. However, neither signals for fear or anxiety nor for neophobia were evidenced by Tg+/– rats, as indicated by the absence of freezing behavior in the 1st exposure to the OF and in the 1st IA session under bright light; and there was not a significant decrease in exploratory activity, either. In addition, time spent in central squares was similar to wt rats and the grooming behavior was also similar (Supplementary Figures 4D–F). There might be different explanations for such discrepancy: (1) We carried out most assays with Tg+/– male rats only and thus, the absence of females and/or Tg+/+ rats could have made the difference—as suggested by homozygous rats behavior in social approach -; (2) The oldest animals we assessed were about 6-month-old, when the signs of anxiety was reported to appear; (3) Our rats were raised in an inverted light-dark cycle with daily manipulations by human operators, and hence, they were assayed during their awaken period, showing far less anxious-like and fear behavior than animals raised in a straight cycle and manipulated at their resting period, as happened in most previous works. These observations claim for comparative studies between both sexes in middle aged animals in similar conditions.

Interestingly, emotional disturbances are a common early symptom in AD patients (Heun et al., 1997; Mizuno et al., 2000). Johansson et al. (2020) reported that apathy and anxiety were associated with Aβ deposition and predicted cognitive decline in humans with mild cognitive impairment (MCI) (Johansson et al., 2020), suggesting that these symptoms may be early clinical manifestations of Alzheimer's disease (Banning et al., 2019; Johansson et al., 2020). In addition, the disruption in preference for social interaction in 4-month-old Tg+/+ homozygous male rats is reminiscent of alterations of social behaviors observed in mouse model of autistic conditions (Moy et al., 2004). Interestingly, βAPP and its metabolites commonly associated with AD, are dysregulated in plasma and brain tissue samples of individuals with Autism Spectrum Disorders (Sokol et al., 2019).

### Synaptic Plasticity-Associated Genes

We examined whether the cognitive deficits observed in 4-month-old transgenic heterozygous male rats would be accompanied by an altered expression of some synaptic plasticity genes in the hippocampus since memory consolidation of the tasks we applied is mainly mediated by this structure, although other central regions might also be involved. For example, STM of object recognition memory depends on the perirhinal cortex for familiarity up to 20 min (Tinsley et al., 2011), though not after 24 h when the hippocampus becomes involved.

It is well-accepted that the transcript levels of synaptic plasticity-associated genes such as *Grin2b* (NR2B), *Gria1* (GluR1), *Arc, Egr1, Homer-1, c-fos, Camk2*β are significantly reduced in the hippocampus of patients at moderate and severe AD stages and of transgenic mouse models of AD, coinciding with extensive amyloid plaque deposition, degeneration and cognitive deficits (Yasuda et al., 1995; Wakabayashi et al., 1999; Sze et al., 2001; Dickey et al., 2003; Hynd et al., 2004; Liang et al., 2008; Wang and Reddy, 2017).

However, there were not significant changes in the basal expression of *Arc, Egr1*, or *c-fos* in the Tg +/– rats up to 6-month-old, while *Dgl4, Syn1*, and *Camk2*β expression was significantly increased in 4-month-old animals only, and *Grin2b* was elevated at both 3 and 4-month-old. As these ages correspond to pre-plaque stages in Tg+/– rats, it supports the idea of an effect dependent on Aβ soluble forms. Reduced expression of immediate early genes like *c-fos* and *Arc*, have been reported in the hippocampus of naïve and memory trained hAPP transgenic mice (Palop et al., 2005; Parra-Damas et al., 2014). Interestingly, Palop et al. (2005) found that basal and activity-induced *Arc* transcript level was reduced particularly in the dentate gyrus of hAPP_FAD_ mice; in this transgenic AD model, exploration of a novel environment significantly increased Arc expression and Arc IR levels in the pyramidal layer and stratum radiatum at CA1, and in the neocortex (as in non-transgenic controls), but not in granule cells of the dentate gyrus (at variance with controls). By comparing different mice lines these authors concluded that *Arc* and *c-fos* expression deficits are influenced by Aβ levels but independently of plaque deposition.

Ca2+/calmodulin (CaM)-dependent protein kinase II beta (CAMK2β) is a protein kinase regulated by the Ca2+/calmodulin complex, that plays a role in LTP, neurotransmitter release and in the regulation of expression of several genes relevant for synaptic and neural plasticity (Blanquet et al., 2003), learning and memory, such as BDNF, NR2B subunit of the glutamate NMDA receptor, syntaxin and acetylcholinesterase. CAMK2β function is primarily associated with its F-actin binding and bundling properties which regulate the proper targeting of CAMK2α to the synapse, rather than with its kinase activity (Borgesius et al., 2011).

Glutamate receptors such as N-methyl-D-aspartate (NMDA) and α-amino-3-hydroxy-5-methyl-4-isoxazolepropionic acid (AMPA) receptors play pivotal roles in synaptic transmission, plasticity, cognition and memory. However, their excessive activation causes excitotoxicity and promotes cell death in a number of neurodegenerative diseases including AD (Guntupalli et al., 2016; Wang and Reddy, 2017). It has been shown that Aβ oligomers binding to or near NMDA receptors inhibits synaptic activity by excessive activation of extrasynaptic NMDA receptors (Decker et al., 2010a), particularly those containing the NR2B subunit, thus promoting cell death pathways (Li et al., 2011), synaptic damage and memory impairment. Aβ oligomers also disrupt neuronal calcium homeostasis implicated in many other neuronal alterations, such as tau hyper-phosphorylation (De Felice et al., 2008), synapse loss (Lacor et al., 2007), and impairment of fast axonal transport (Decker et al., 2010b; Kim et al., 2011; Torres et al., 2012).

We showed that transcripts for NR2B were elevated in 4-month-old Tg+/– compared to wt rats while other subunits transcripts did not change. This might lead to an imbalance between NMDA receptors subunits, which in turn, could promote cell death pathways (Li et al., 2011). However, although *Grin2b* transcript levels were higher in 3- and 4-month-old Tg+/– rats, NMDA receptor subunits levels were not significantly higher in 4-month-old Tg+/– compared to wt rats. Several studies indicate that mRNA and protein levels of NMDA and AMPA receptors subunits are reduced in moderate and severe AD (Yasuda et al., 1995; Hynd et al., 2001, 2004; Sze et al., 2001), and that synaptic scaffold proteins SYN-1 and PSD-95 are also reduced in AD and in hAPP transgenic mice (Pham et al., 2010; Shao et al., 2011). SYN-1 is a pre-synaptic protein that responds to neuronal activity and regulates synaptic vesicle trafficking for neurotransmitter release (Marsh et al., 2017). PSD-95 clusters glutamate receptors and the associated synaptic signaling pathways, being also involved in the morphology and strength of synapses (Matsuzaki et al., 2004; Chen et al., 2011, 2015). Although seemingly counter intuitive, the upregulation of transcript levels of both *Grin1* and *Grin2b* in 3-month-old male Tg+/– rats as well as that of *Grin2b, Dlg4, Camk2b*, and *Syn1* in 4-month-old Tg+/– rats, together with an increase of PSD-95 and CAMK2β protein levels at these early, pre-plaque stages, is not unexpected. An increasing number of studies looking at early stages of AD in post-mortem human brain tissue and in animal models support the concept of differential brain gene expression in pre-symptomatic and pathological AD stages (Bossers et al., 2010; Berchtold et al., 2014), reviewed in Saura et al. (2015). Bimodal changes in brain activity at pre-symptomatic and clinical AD stages have also been revealed by functional imaging studies (Sperling, 2011). As an example, prior to the deposition of plaques, 3xTg-AD mice show enhanced synaptic plasticity which is accompanied by early hippocampus-dependent learning/memory impairments (Davis et al., 2014). This coincides with increased expression of synaptic plasticity-related genes such as *Egr1, Snap25, Gria1, Gria3*, and *Gria4* (Cantanelli et al., 2014; Gatta et al., 2014). The overexpression of transcripts encoding for proteins involved in synaptic plasticity suggests an excitatory response to the accumulation of amyloid peptides. It may also be interpreted as a transient protective mechanism against amyloid-driven synaptic dysfunction, as previously suggested in a study using 3xTg-AD mice (Baazaoui et al., 2017). It might also coincide with the paradoxical upregulation of presynaptic boutons as seen in MCI and in the McGill-Thy1-APP mouse model of AD (Bell et al., 2003, 2007). In addition, the fact that the increase in the expression of *Grin2b, Dlg4, Camk2b*, and *Syn1* does not translate into a significant increase in the transcription of immediate early genes such as *c-fos, Arc*, and *Egr1* in Tg+/– rats suggests a disconnection in the signaling pathways connecting CAMK2β to cAMP-response element (CRE)-directed gene expression. We have previously demonstrated *in vitro* a similar Aβ-driven disconnection between RAP1/MEK/ERK activation and CRE-directed gene expression, possibly mediated by the sequestration of CBP (Echeverria et al., 2005).

We have also reported that the pathological accumulation of intracellular Aβ leads to impairments in the nuclear mobilization of both CREB (Arvanitis et al., 2007) and of CRTC1 (Wilson et al., 2016). In both situations this impairment resulted in diminished activation of CRE-regulated gene expression responsible for the generation of key synaptic plasticity-related proteins, including *Arc, c-fos*, and *Egr1*. Of note, these observations were made in presence of high levels of intracellular Aβ: expression *in vitro* of hAPP driven by a strong promoter CAMKII-tTA or in homozygous McGill-R-Thy1-APP rats. Therefore, we can speculate that the low levels of intracellular Aβ in Tg+/– at these early time-points would be sufficient to generate an increase in the expression of the synaptic genes *Grin2b, Dlg4, Syn1*, and in *Camk2*β but not to provoke a significant reduction in the levels of CRE-regulated synaptic genes such as *c-fos, Arc*, and *Egr1*. Such hypothesis is strengthen by the fact that in APP_Sw,Ind_ mice, akin to our observations in homozygous McGill-R-Thy1-APP rats (Wilson et al., 2016), a dysregulation of the CREB/CRTC1-regulated transcriptional program leads to reduced expression of *c-fos* and *Arc* at 6-months, but not at 2-months of age (Parra-Damas et al., 2014). Alternatively, an early reduction in the expression of these genes might be obscured by the fact that the transcripts originated from several neuronal populations. Indeed, it was reported that *Arc* expression was affected by the presence of hAPP and Aβ peptides in dentate granule cells but not in other neuronal populations, suggesting a regional vulnerability (Palop et al., 2005). Whether the increases in gene expression here reported are directly related with the cognitive deficits and behavioral alterations observed in this Tg rat model deserve further attention.

In conclusion, our findings provide novel information on the cognitive deficits present in McGill-R-Thy1-APP Tg rats. Having into account that sex impacts the AD pathological process (Ferretti et al., 2018), our assays were exclusively developed in Tg males -as some preliminary results indicate that there are significant differences between females and males performance in some paradigms (Habif et al., 2019). The fact that the deficits in LTM and in social interaction were observed in Tg+/– rats at early stages of AD-like brain amyloidosis, with intracellular Aβ accumulation but in the absence of plaques, further highlights the importance of Aβ oligomers and/or other APP-fragments in behavior and memory processes. It also emphasizes the validity of the McGill-R-Thy1-APP Tg rat to study AD-like early processes. Remarkably salient and potentially predictive features of this animal model are based on the already well-established alterations -cognitive deficits, plasticity genes changes and synaptic plasticity inhibition- at very early stages, suggesting that similar changes might also be present in preclinical AD stages, though unnoticed due to the neural reserve -and the associated cognitive reserve- of the human beings.

## Data Availability Statement

The raw data supporting the conclusions of this article will be made available by the authors, without undue reservation.

## Ethics Statement

The animal study was reviewed and approved by Comité Institucional para el Cuidado y Uso de Animales de Laboratorio (CICUAL), Facultad de Medicina, Universidad de Buenos Aires - Argentina. https://www.fmed.uba.ar/cicual/informacion-general.

## Author Contributions

MH, SDC, MB, and DJ conceived the study and designed the experiments. MH, SDC, MB, NC, MCC, DS, MA, CS, VB, MPC, and TG performed the experiments and collected the data. MH, SDC, MB, NC, ACC, and DJ analyzed and interpreted the results. MH, SDC, MB, and DJ designed the figures. MH, SDC, MB, NC, and DJ drafted the manuscript. SDC, ACC, and DJ wrote the final version of the manuscript. DJ coordinated the whole project. All authors revised the manuscript, contributed to the article, and approved the submitted version.

## Conflict of Interest

The authors declare that the research was conducted in the absence of any commercial or financial relationships that could be construed as a potential conflict of interest. The handling Editor declared a shared affiliation, though no other collaboration, with several of the authors MH, MB, NC, MC, DS, MA, CS, VB, MPC, TG, DJ.
