# Supplementary material for: Early Long-Term Memory Impairment and Changes in the Expression of Synaptic Plasticity-Associated Genes, in the McGill-R-Thy1-APP Rat Model of Alzheimer's-Like Brain Amyloidosis
Source: Front Aging Neurosci. 2021 Jan 22;12:585873. doi: 10.3389/fnagi.2020.585873 (PMC7862771; doi:10.3389/fnagi.2020.585873)
Supplement: Supplementary file 1 [file Image_1.pdf]

Supplementary Figure 1 (S1)

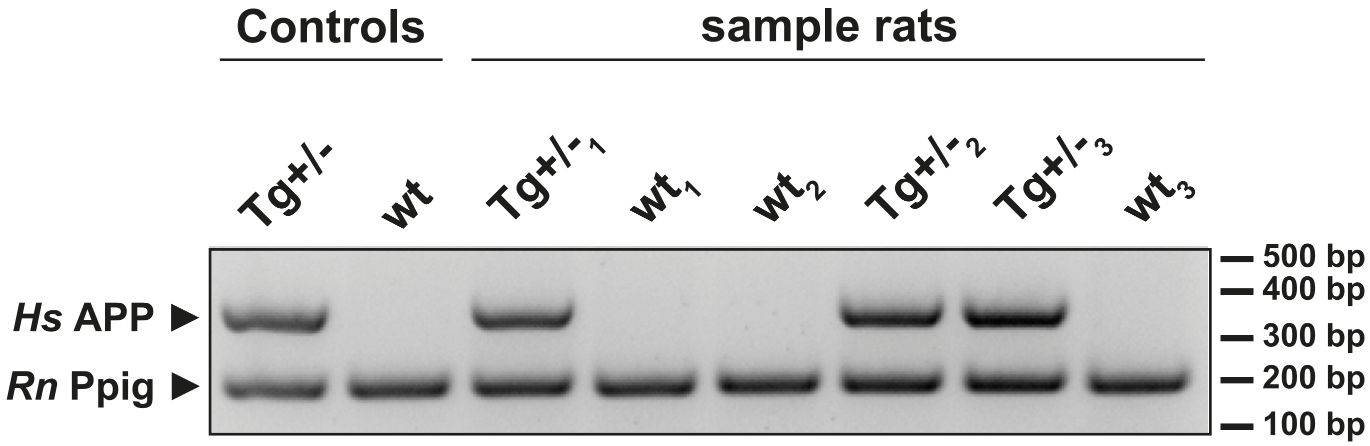

Supplementary Figure 1 (S1). Genotyping of McGill-R-Thy1-APP Heterozygous Tg rats. Tg+/- rats were identified following PCR by the presence of a 377bp amplicon from NM_000484.4 - Homo sapiens amyloid beta precursor protein - hAPP - transgene. The lower band present in both wt and Tg+/- rats corresponds to the 207bp amplicon from XM_006234324.3 - Rattus norvegicus peptidylprolyl isomerase G - Ppig – gene, used as an internal control. DNA from rats previously genotyped and characterized was used as positive and negative controls. A representative set of samples is shown.
