# Supplementary material for: Early Long-Term Memory Impairment and Changes in the Expression of Synaptic Plasticity-Associated Genes, in the McGill-R-Thy1-APP Rat Model of Alzheimer's-Like Brain Amyloidosis
Source: Front Aging Neurosci. 2021 Jan 22;12:585873. doi: 10.3389/fnagi.2020.585873 (PMC7862771; doi:10.3389/fnagi.2020.585873)
Supplement: Supplementary file 2 [file Image_2.pdf]

Supplementary Figure 2 (S2)

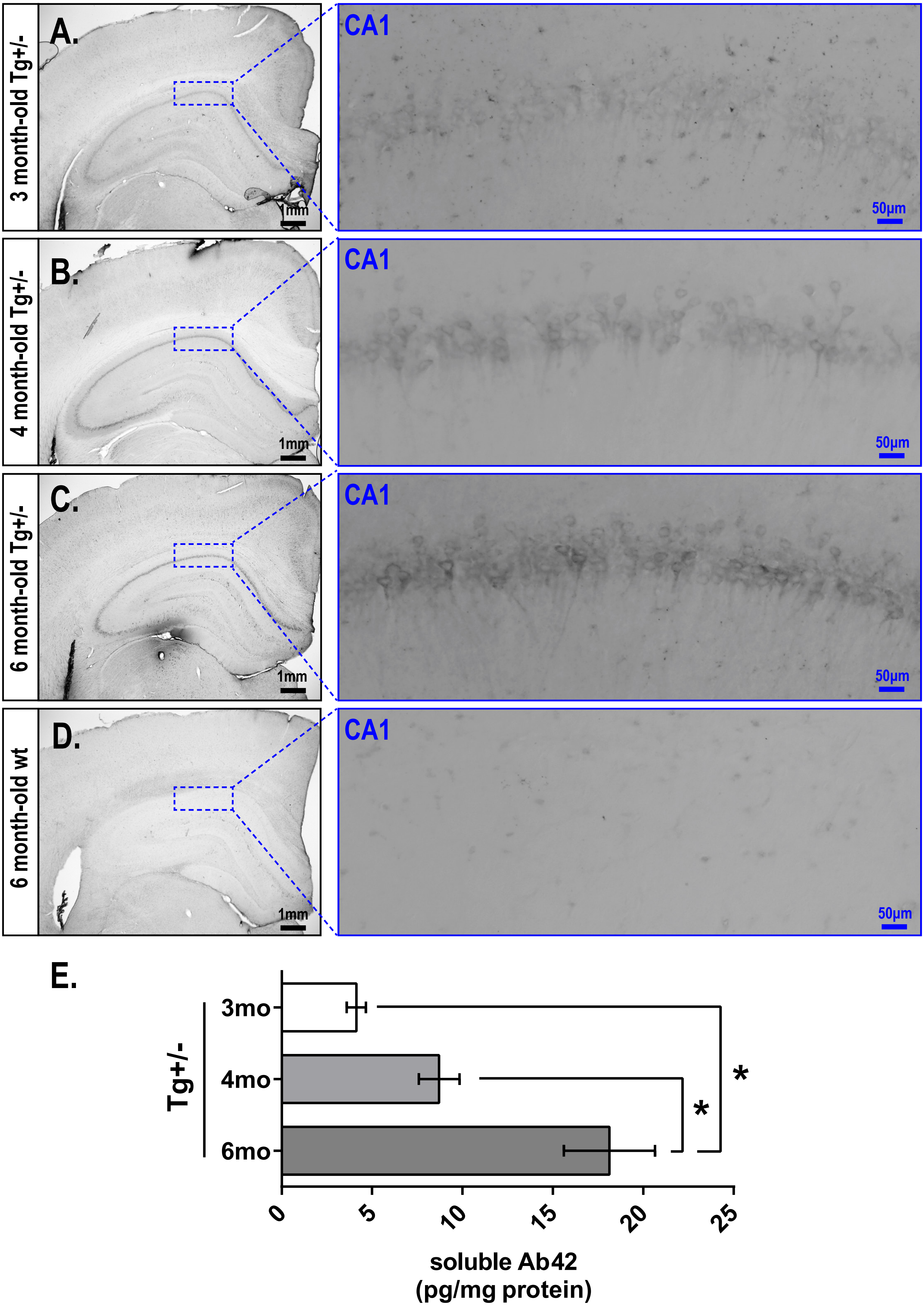

Supplementary Figure 2 (S2). Pre-plaque amyloid pathology in McGill-R-Thy1-APP Tg+/- rats. A-D) Representative pictures of human Aβ immunoreactivity detected using the monoclonal antibody McSA1 at 3 **(A)**, 4 **(B)** and 6 **(C)** months of age. At early stages of amyloid pathology, intraneuronal Aβ accumulates in the cerebral cortex and hippocampus (scale bar 1mm). As expected, wild-type rats **(D)** are devoid of McSA1 immunoreactivity. Magnified views of CA1 regions are included as insets at the right side of each brain slice picture, clearly showing intraneuronal Aβ accumulation (scale bar 50μm). **E)** Abundance of TBS-soluble human Aβ42 peptides in the hippocampus was determined by ELISA (NTg+/-=4-5 rats per age). Data are expressed as mean ± SEM. *$p < 0.05$, analyzed with a two-tailed, unpaired t test.
