# Supplementary material for: Early Long-Term Memory Impairment and Changes in the Expression of Synaptic Plasticity-Associated Genes, in the McGill-R-Thy1-APP Rat Model of Alzheimer's-Like Brain Amyloidosis
Source: Front Aging Neurosci. 2021 Jan 22;12:585873. doi: 10.3389/fnagi.2020.585873 (PMC7862771; doi:10.3389/fnagi.2020.585873)
Supplement: Supplementary file 3 [file Image_3.pdf]

# Supplementary Figure 3 (S3)

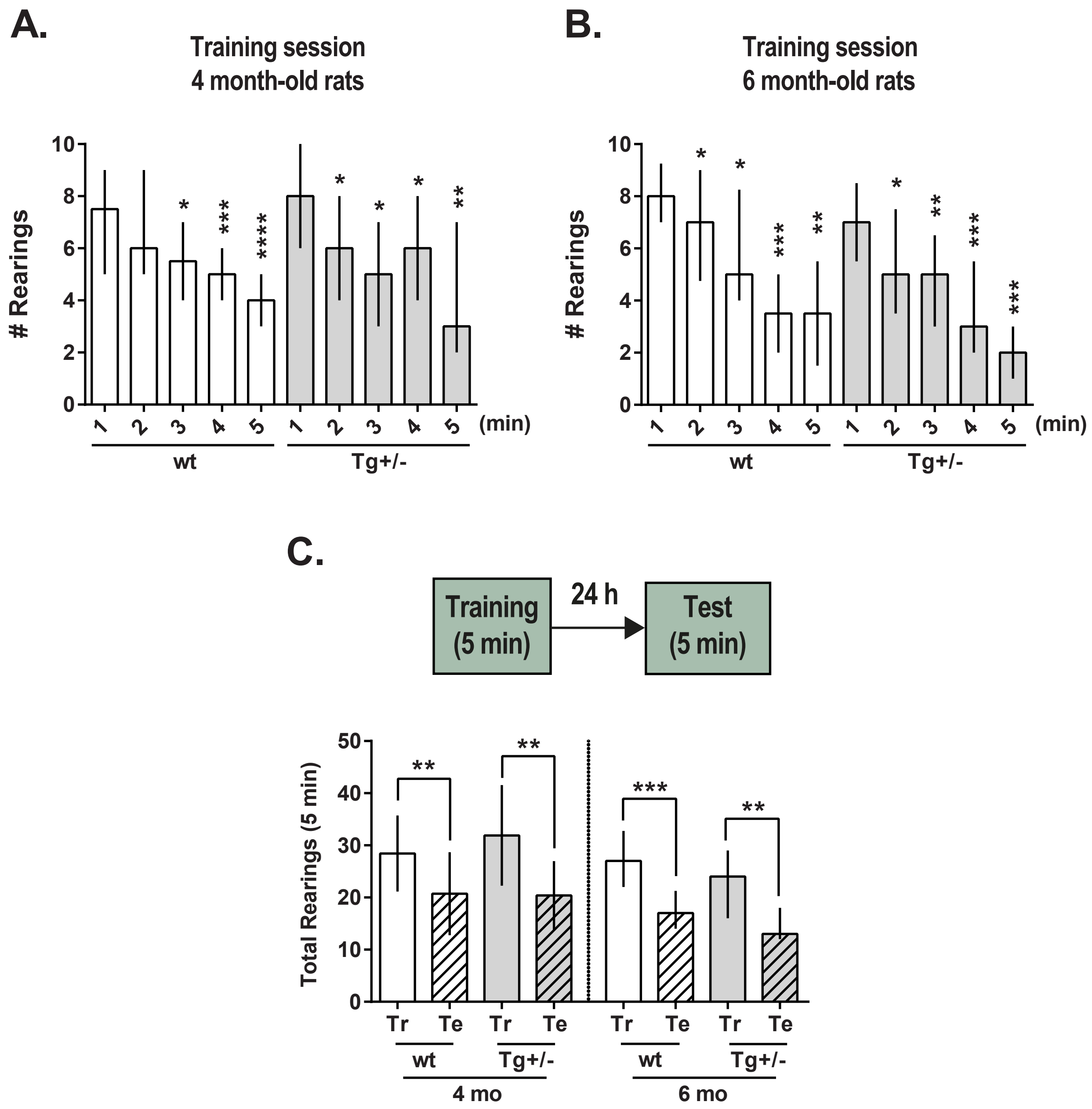


**Supplementary Figure 3 (S3). Short and long term habituation (ST, LT) to an OF. Number of rearings (vertical exploration) in 5 minutes sessions.** McGill-R-Thy1-APP Tg+/- male rats (dark gray bars) and their wt littermates (white bars) were trained and tested for **ST** (intra-session) **habituation to an OF at A) 4** and **B) 6 months** of age. Bar diagram shows median and interquartile ranges (25;75) for the number of rearings recorded during each minute of a 5 minute session (training, Tr). Significant differences were assessed by Wilcoxon matched-pairs signed rank test; * P<0.05, ** P<0.01, *** P<0.001, **** P<0.0001. **D) LT habituation to an OF (LTM)**. Bar diagram shows median and interquartile ranges (25;75) for the total number of rearings recorded during a 5 minutes training session (Tr, plain bars) and during the test session (Te, stripped bars) which was performed 24hr later (schematic representation of the task steps on top). No significant differences were observed between any selected pair of training sessions amongst the wt or Tg+/- animals (Unmatched Kruskal-Wallis test with Dunn's multiple comparisons). Total rearings during test session were significantly lower than during training session for the two different ages, either for wt or Tg animals. Statistically significant differences were assessed by Wilcoxon matched-pairs signed rank test, (** P<0.01, *** P<0.001. (4mo: $N_{wt}$=12, $N_{Tg+/-}$=12; 6mo: $N_{wt}$=12, $N_{Tg+/-}$=12).
