# Supplementary material for: Early Long-Term Memory Impairment and Changes in the Expression of Synaptic Plasticity-Associated Genes, in the McGill-R-Thy1-APP Rat Model of Alzheimer's-Like Brain Amyloidosis
Source: Front Aging Neurosci. 2021 Jan 22;12:585873. doi: 10.3389/fnagi.2020.585873 (PMC7862771; doi:10.3389/fnagi.2020.585873)
Supplement: Supplementary file 4 [file Image_4.pdf]

# Supplementary Figure 4 (S4)

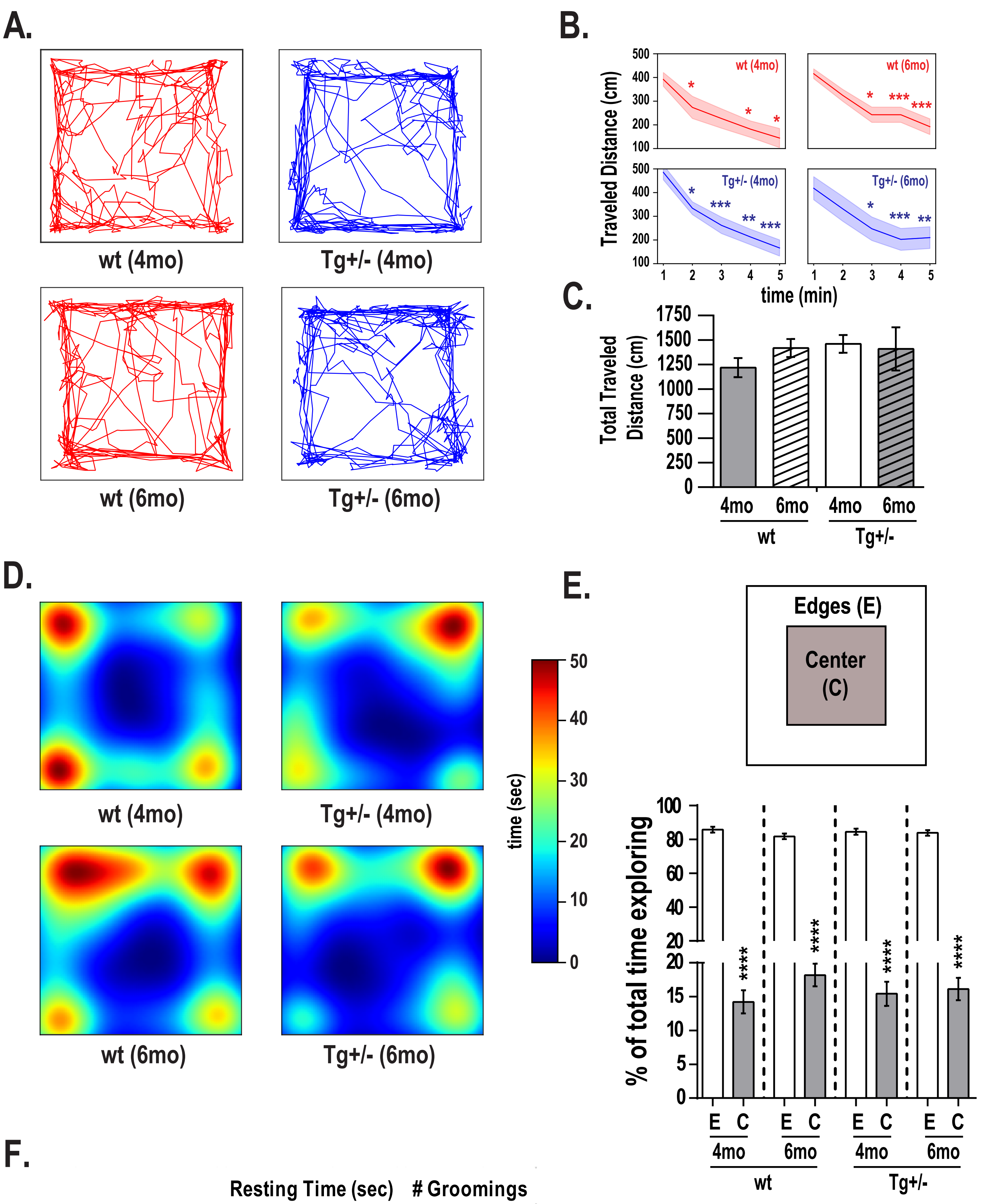


F.

| | | Resting Time (sec) | # Groomings |
|---|---|---|---|
| wt | 4 mo | 11,88 ± 3,56 | 1,75 ± 0,49 |
| | 6 mo | 11,50 ± 4,03 | 1,83 ± 0,47 |
| Tg+/- | 4 mo | 18,75 ± 6,67 | 2,50 ± 0,56 |
| | 6 mo | 22,38 ± 4,20 | 2,25 ± 0,52 |

**Supplementary Figure 4 (S4). Quantitative analysis of trajectory, traveled distance and resting time in the OF: A) Overall trajectory in the experimental arena.** Path traveled by McGill-R-Thy1-APP Tg+/- male rats (blue line) and their wt littermates (red line) during the 5 minutes training session (Tr). Representative trajectories for 4- and 6-month-old wt ad Tg+/- rats. **B) Traveled distance based on time segment.** Average distance traveled by Tg+/- male rats (blue line) and their wt littermates (red line) was calculated in one-minute bins. Numbers represent mean traveled distance ± SEM. Statistically significant differences - indicating short term habituation (ST) to the OF- were assessed by One-way ANOVA, Dunnett post-hoc test (*, $P<0.05$, ** $P<0.01$, *** $P<0.001$). **C) Total distance traveled**. Bar diagram represents mean distance traveled ± SEM in the total 5 minutes time-frame. No statistically significant differences were observed for any selected pair of genotype and age (One-way ANOVA, Dunnett post-hoc test). **D) Heat-map plot indicating time spent in the different locations**. For the raw scatter plot of the rat position (one xy-coordinate per time-point), the plotting window was cut in several bins, and the number of data points in each bin is represented by a different color. The resulting 2D histogram was subjected to a Gaussian KDE (kernel density estimate) processing to generate the final smoothed 2D density plot. Red = more time, blue = less time. Representative heat-maps for 4- and 6-month-old. wt and Tg+/- rats. **E) Bidimensional exploratory behavior by zone**. Two regions-of-interest (ROIs), the outer edge (E, white) and the center (C, gray) of the arena were delimitated. Number of time-points within each ROI were counted and expressed as a percentage of the 5 minutes time-frame total time-points. Bars represent mean exploration time ± SEM. Time spent in (C) was significantly lower compared with time in (E) for any selected genotype and age (**** $P<0.0001$, One-way ANOVA, Tukey´s multiple comparisons test). No statistically significant differences were observed between time spent in (E) among the different groups, and (C) values were also similar between groups (One-way ANOVA, Tukey´s multiple comparisons test). **F) Resting time and stereotyped behavior**. The amount of time the rats remained motionless as well as the repetitive movement (number of *grooming stereotypies*) were assessed. Numbers represent mean values ± SEM. No statistically significant differences in these parameters were observed for any selected pair of genotype and age (One-way ANOVA, Dunnett post-hoc test).
