# Supplementary material for: Early Long-Term Memory Impairment and Changes in the Expression of Synaptic Plasticity-Associated Genes, in the McGill-R-Thy1-APP Rat Model of Alzheimer's-Like Brain Amyloidosis
Source: Front Aging Neurosci. 2021 Jan 22;12:585873. doi: 10.3389/fnagi.2020.585873 (PMC7862771; doi:10.3389/fnagi.2020.585873)
Supplement: Supplementary file 5 [file Image_5.pdf]

Supplementary Figure 5 (S5)

A.

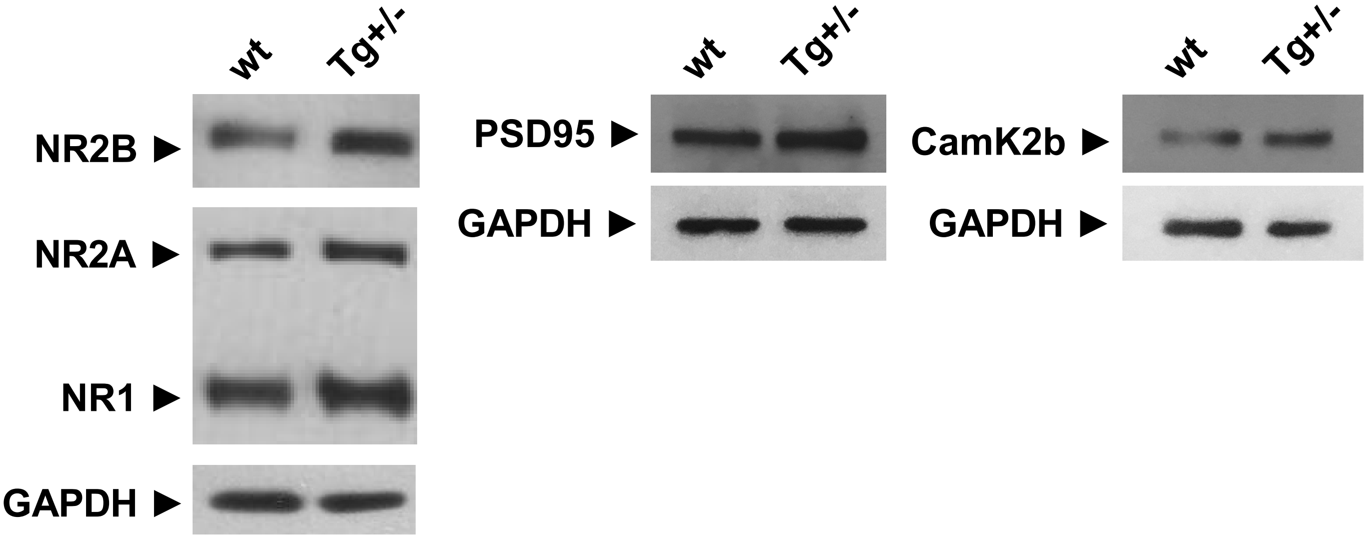

B.

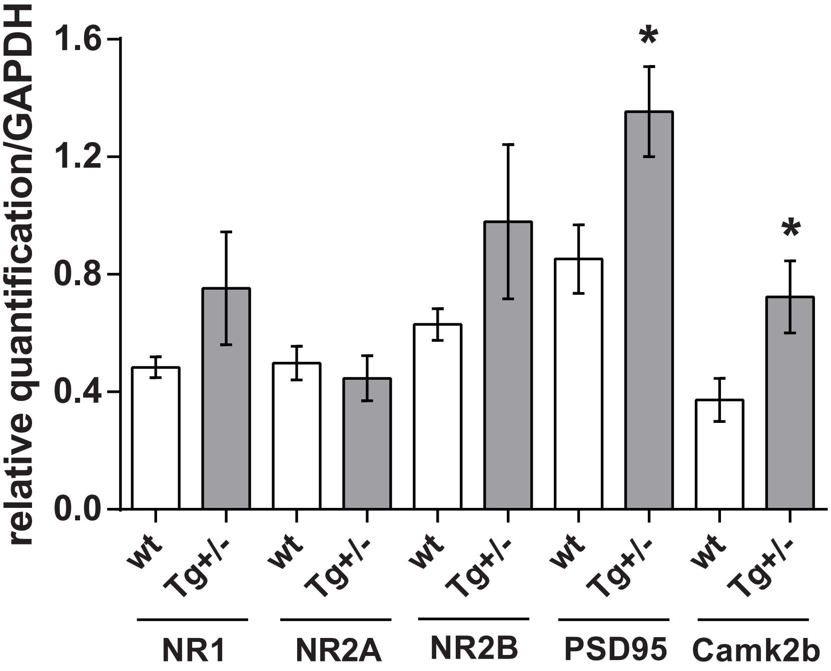

Supplementary Figure 5 (S5). Basal expression levels of NMDAr subunits and synaptic proteins in McGill-R-Thy1-APP Tg+/- and wt rats. A) Western blotting was performed on hippocampal lysates from 4-month-old Tg+/- (NTg+/-=5) and wt rats (Nwt=4-5) and probed for NR1, NR2A, NR2B, PSD-95 and CAMK2β protein levels as well as GAPDH as loading control. **B)** Quantification of the protein levels by densitometry. Values are mean ± SEM. *p < 0.05, analyzed with a two-tailed, unpaired t test.
